# Structural insights into the Bre1–Lge1 and RNF20/RNF40–WAC interactions critical for H2B ubiquitination

**DOI:** 10.1093/nar/gkaf1514

**Published:** 2026-01-14

**Authors:** Meng Shi, Xuejie Wang, Hang Zhang, Yajiao Wen, Qianqian Liu, Pu Chen, Xuefeng Chen, Song Xiang

**Affiliations:** Department of Biochemistry and Molecular Biology, Key Laboratory of Immune Microenvironment and Disease (Ministry of Education), The province and ministry co-sponsored collaborative innovation center for medical epigenetics, State Key Laboratory of Experimental Hematology, Tianjin Medical University, Tianjin 300070, P.R. China; Department of Nephrology, Children’s Hospital of Chongqing Medical University, National Clinical Research Center for Children and Adolescents' Health and Diseases, Ministry of Education Key Laboratory of Child Development and Disorders, Chongqing Key Laboratory of Pediatric Metabolism and Inflammatory Diseases, Genome Stability and Pediatric Cancer Center, Chongqing 400015, P.R. China; State Key Laboratory of Microbial Technology, Shandong University, Qingdao 266237, P.R. China; Department of Biochemistry and Molecular Biology, Key Laboratory of Immune Microenvironment and Disease (Ministry of Education), The province and ministry co-sponsored collaborative innovation center for medical epigenetics, State Key Laboratory of Experimental Hematology, Tianjin Medical University, Tianjin 300070, P.R. China; Department of Biochemistry and Molecular Biology, Key Laboratory of Immune Microenvironment and Disease (Ministry of Education), The province and ministry co-sponsored collaborative innovation center for medical epigenetics, State Key Laboratory of Experimental Hematology, Tianjin Medical University, Tianjin 300070, P.R. China; Department of Biochemistry and Molecular Biology, Key Laboratory of Immune Microenvironment and Disease (Ministry of Education), The province and ministry co-sponsored collaborative innovation center for medical epigenetics, State Key Laboratory of Experimental Hematology, Tianjin Medical University, Tianjin 300070, P.R. China; Department of Nephrology, Children’s Hospital of Chongqing Medical University, National Clinical Research Center for Children and Adolescents' Health and Diseases, Ministry of Education Key Laboratory of Child Development and Disorders, Chongqing Key Laboratory of Pediatric Metabolism and Inflammatory Diseases, Genome Stability and Pediatric Cancer Center, Chongqing 400015, P.R. China; Department of Biochemistry and Molecular Biology, Key Laboratory of Immune Microenvironment and Disease (Ministry of Education), The province and ministry co-sponsored collaborative innovation center for medical epigenetics, State Key Laboratory of Experimental Hematology, Tianjin Medical University, Tianjin 300070, P.R. China

## Abstract

The mono-ubiquitination of the histone protein H2B (H2BUb1) has important functions in transcription, DNA repair, and other chromatin-related processes. The reaction is catalyzed by Bre1 and the homologous RNF20/RNF40 complex in the budding yeast and human cells, respectively, and is promoted by their respective interaction partners, Lge1 and WAC. The mechanism of the Bre1–Lge1 and RNF20/RNF40–WAC interactions is poorly understood. Here, we present the crystal structure of the Bre1–Lge1 complex and an AlphaFold predicted structure model of the RNF20/RNF40 complex bound with WAC, as well as *in vitro* and *in vivo* experiments to assess the interaction mechanism and function. Our study revealed extensive Bre1–Lge1 and RNF20/RNF40–WAC interfaces and a structural homology shared by these interfaces, but completely different sets of key electrostatic interactions at these interfaces that are crucial for the binding and encode the binding specificity. We further found that these interactions play critical roles in the Bre1-catalyzed H2BUb1 reaction and processes it regulates. Our data provide insights into the mechanism of the Bre1–Lge1 and RNF20/RNF40–WAC interactions.

## Graphical Abstract

**Graphical Abstract:**
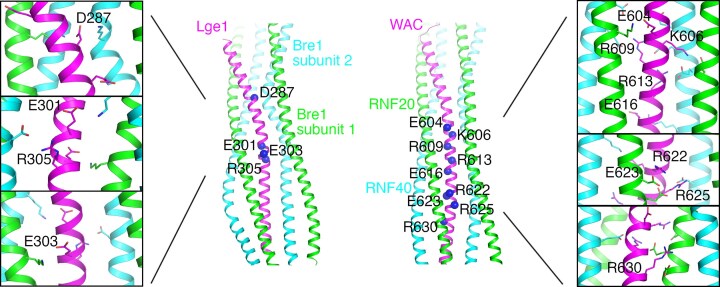

## Introduction

Post-translational modifications on the nucleosome, the basic unit of chromatin, play critical roles in coordinating genome-related processes in eukaryotes [1–4]. The nucleosome is composed of ∼150 bp of DNA double strand wrapped around a histone protein core complex that typically contains two copies of H2A, H2B, H3, and H4 [5]. Ubiquitination of H2B has been found in yeast and human cells, which primarily attaches one ubiquitin molecule to a highly conserved lysine residue in the H2B C-terminal helix (Lys123 in the budding yeast, Lys120 in human cells, H2BUb1) [6, 7]. The H2BUb1 modification has been found to regulate several important chromatin-related processes [8, 9]. It is associated with actively transcribed genes [10–12] and is coupled to RNA polymerase II elongation rate [13]. It promotes gene transcription by inducing an open and accessible chromatin conformation [14], coordinating the function of the FACT histone chaperone complex [15–18], and signaling for H3K4 and H3K79 methylation [19–23]. In line with the latter function, recent structural studies revealed a role of the H2B-attached ubiquitin in recruiting and activating the COMPASS complex and DOT1L that catalyze the H3K4 and H3K79 methylation reactions, respectively [24–30]. It also regulates several important DNA damage responses, including checkpoint activation [31, 32], homologous recombination (HR), non-homologous end-joining repair of DNA double-strand breaks (DSBs) [32–34], and translesion synthesis [35]. During meiosis, it is crucial for the programmed DSB repair in meiotic recombination [36, 37]. Additional processes it regulates include nucleosome positioning, chromatin segregation, RNA processing, and replication [8]. In line with these important functions, the H2BUb1 modification is implicated in several types of cancer [38, 39].

The ubiquitination reaction requires several enzymes. The ubiquitin-activating enzyme (E1) conjugates ubiquitin to the ubiquitin-conjugating enzyme (E2); the ubiquitin ligase (E3) subsequently transfers ubiquitin from the ubiquitin–E2 conjugate to the substrate. Most organisms contain a few and a few dozen E1 and E2 enzymes, respectively, but several hundred E3 enzymes that encode the substrate specificity [40, 41]. In the budding yeast, the E3 enzyme Bre1 together with the E2 enzyme Rad6 catalyzes the H2BUb1 reaction [7, 42, 43]. Homologs of Bre1 and Rad6 are widely found in eukaryotic organisms [44]. In human cells, the Bre1 homologs RNF20 and RNF40 form a heterodimer and catalyze the H2BUb1 reaction together with the human Rad6 homologs [45–47]. The substrate specificity of Bre1 and the RNF20/RNF40 complex is partly contributed by their RING domains. In addition to promoting the active “closed” conformation of the ubiquiti∼Rad6 conjugate [48], these RING domains also interact with the nucleosome to position the ubiquitin–Rad6 conjugate for the ubiquitin transfer [49–52]. The H2BUb1 reaction also requires Bre1’s N-terminal Rad6 binding domain (RBD) that binds Rad6 and coordinates with the RING domain during catalysis [53–55]. The N-terminal regions in RNF20 and RNF40 probably have a similar function [47].

The H2BUb1 reaction in budding yeast and human cells requires Lge1 [56] and WAC [57], respectively. Lge1 binds to Bre1 and facilitates Bre1 recruitment to the chromatin [42, 56]. It was recently found that the intrinsically disordered region (IDR) in Lge1 promotes liquid–liquid phase separation (LLPS), and the resulting Lge1 condensates recruit Bre1, Rad6, and the nucleosome to accelerate the H2BUb1 catalysis [58]. WAC also binds to the RNF20/RNF40 complex and facilitates its recruitment to actively transcribed genes [57]. It bears little sequence homology to Lge1 but also contains an IDR that can compensate for the Lge1 IDR deletion in budding yeast cells, suggesting that it may also promote LLPS formation to stimulate the H2BUb1 catalysis [58].

**Figure 1.**
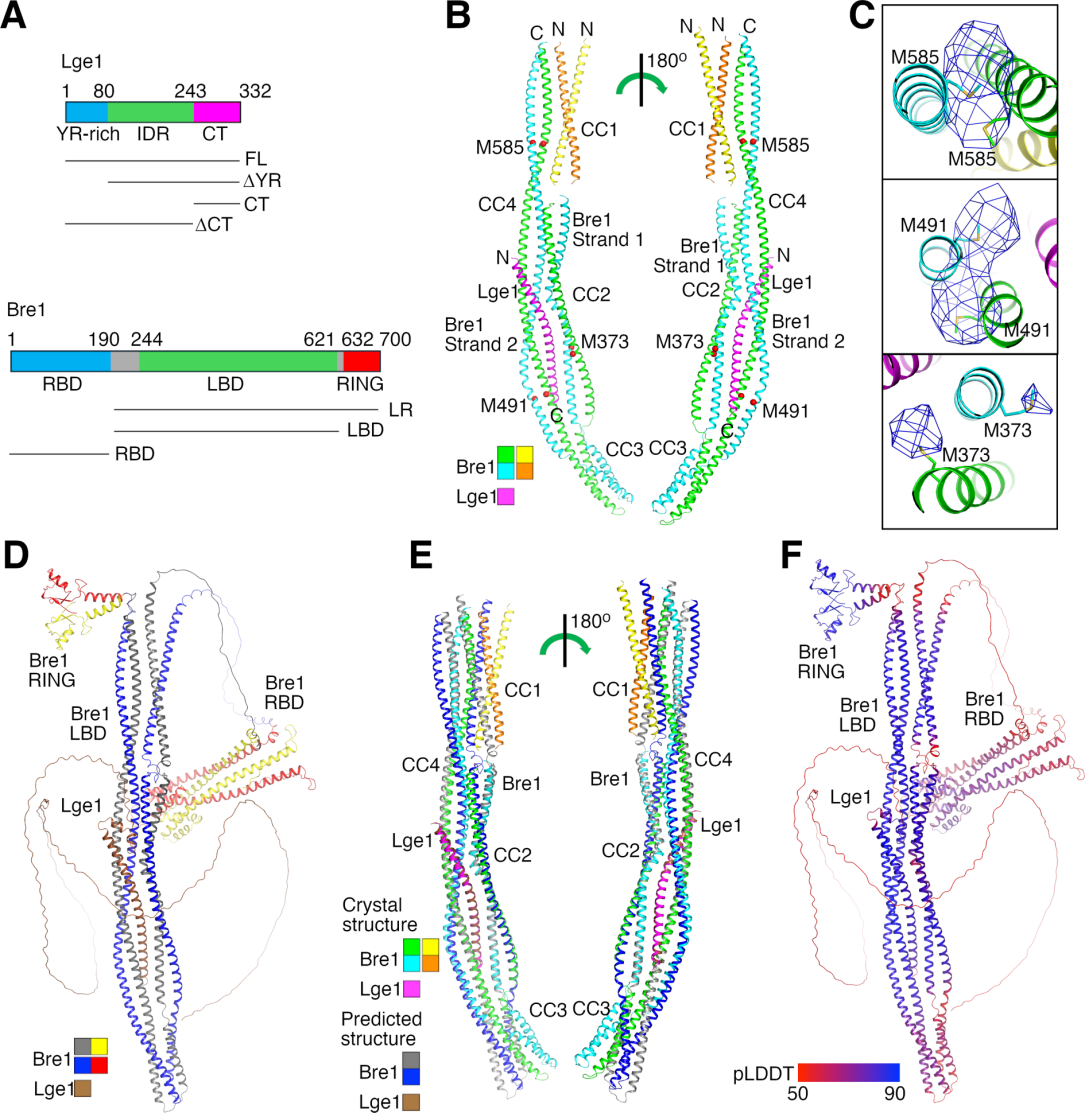
Structure of the Bre1–Lge1 complex. (**A**) Domain architecture of Lge1 and Bre1. Lge1 and Bre1 fragments used in this study are indicated. YR-rich, tyrosine and arginine-rich region. (**B**) Crystal structure of the Bre1-LR–Lge1-CT complex. The two polypeptides in Bre1 and Lge1 are colored in green, cyan, and magenta, respectively. The two polypeptides in Bre1 CC1 are colored in yellow and brown. The linker between CC1 and CC2 is disordered in the structure, making it impossible to determine how polypeptides in CC1 and CC2 are connected. The three Met residues in Bre1–LBD are indicated with red spheres. The coloring scheme is used throughout the paper unless otherwise indicated. (**C**) Difference anomalous densities associated with selenium in SeMet. The density was contoured at 2.5 σ. The Met residues are shown for reference. (**D**) The AlphaFold predicted structure of the Bre1–Lge1 complex. (**E**) Superimposition of the predicted and crystal structures. For clarity, only Lge1-CT and Bre1 LBD in the predicted structure are shown. (**F**) PLDDT scores of residues in the predicted structure. Structure figures were produced with PyMOL (https://pymol.org).

The physiological importance of the Bre1–Lge1 and RNF20/RNF40–WAC interactions is highlighted by the fact that deleting Lge1 or WAC severely inhibited H2BUb1 production in the budding yeast or human cells, respectively [56, 57]. It has been reported that the C-terminus of Lge1 (Lge1-CT) and WAC (WAC-CT), both predicted to mediate coiled-coil interactions, interact with the middle regions in Bre1 (Lge1 binding domain, LBD, Fig. 1A) and the RNF20/RNF40 complex, respectively [57, 58]. Due to a lack of structural information, the mechanism of these interactions and how they contribute to the H2BUb1 catalysis are poorly understood. Here, we present crystal structure of the Bre1–Lge1 complex and an AlphaFold-predicted structure model of the RNF20/RNF40 complex bound with WAC, as well as *in vitro* and *in vivo* experiments to probe the interaction mechanism and function. The structures revealed extensive Bre1–Lge1 and RNF20/RN40-WAC interfaces and a structural homology shared by these interfaces. Structure-guided interaction studies revealed completely different sets of key electrostatic interactions at these interfaces, which are crucial for the binding and encode the binding specificity. We further found that these key interactions are crucial for the Bre1-catalyzed H2BUb1 reaction and its cellular function. Our data provide insights into the mechanism of the Bre1–Lge1 and RNF20/RNF40–WAC interactions and their function.

## Materials and methods

### Protein expression and purification

To purify the Bre1-LR–Lge1-CT complex, gene fragments encoding residues 227–700 in Bre1 and 261–332 in Lge1 were inserted into vectors pET26B (Novagen) and pTXB1 (New England Biolabs), respectively. The recombinant Bre1-LR and Lge1-CT contain no tag and an intein-chitin binding domain (CBD) tag, respectively. The plasmids were transformed separately to *Escherichia coli* BL21 Rosetta (DE3) cells. The cells were induced with 0.25 mM isopropyl β-d-1-thiogalactopyranoside (IPTG, Bio Basic) for 16 h at 16°C, mixed, and lysed with an AH-2010 homogenizer (ATS Engineering). The Bre1–Lge1 complex was purified from the cleared cell extract with chitin (New England Biolabs), heparin (Cytiva), and gel filtration (Superose 6 10/300, Cytiva) columns. The purified complex was concentrated to 10 mg/ml in a solution containing 20 mM TRIS (pH 7.5), 200 mM sodium chloride, and 2 mM dithiothreitol (DTT), flash-frozen in liquid nitrogen, and stored at −80°C.

The selenomethionine (SeMet)-substituted Bre1-LR was expressed by inhibiting the methionine synthesis and supplementing SeMet [59]. The purification of the Bre1-LR–Lge1-CT complex containing SeMet-substituted Bre1-LR was the same as the purification of the native complex, except that the DTT concentration was increased to 10 mM.

To purify Bre1 and its fragments, the *BRE1* gene or fragments encoding its RBD (residues 1–220) or LR (residues 227–700) regions were inserted into vector pET26b. The recombinant proteins contain a 6× histidine tag. The proteins were expressed in *E. coli* BL21 Rosetta (DE3) cells. Bre1 RBD was purified with the nickel–nitrilotriacetic acid agarose (Ni-NTA, Smart Life Sciences) column, and Bre1 and Bre1-LR were purified with Ni-NTA, heparin, and gel filtration (Superdex 75 increase 10/300, Cytiva) columns. The proteins were concentrated to concentrations of 8–15 mg/ml in a solution containing 20 mM TRIS (pH 7.5) and 200 mM sodium chloride, flash-frozen in liquid nitrogen, and stored at −80°C.

Lge1 was purified as described [58]. Briefly, recombinant Lge1 with a 6× histidine tag was expressed in *E. coli* BL21 Rosetta (DE3) cells. The cell extract was incubated in a denaturing solution [10 mM TRIS (pH 8.0), 100 mM sodium phosphate monbasic, 8 M urea] with Ni-NTA. After removing urea by extensive washing, Lge1 was eluted with a buffer containing 10 mM TRIS (pH 7.2), 1 M sodium chloride, and 1 M imidazole, concentrated to 5 mg/ml, flash-frozen in liquid nitrogen, and stored at −80°C. Lge1-CT was purified from *E. coli* BL21 Rosetta (DE3) cells harboring the pTXB1-derived plasmid for Lge1-CT with chitin, heparin, and gel filtration (Superdex 75 increase 10/300) columns, concentrated to 0.8 mg/ml in a solution containing 20 mM TRIS (pH 7.5) and 200 mM sodium chloride, flash-frozen in liquid nitrogen, and stored at −80°C.

The budding yeast E1 enzyme Uba1 and ubiquitin were purified as described [54, 60, 61]. Briefly, Uba1 (residues 10–1024) and ubiquitin were expressed in *E. coli* BL21 Rosetta (DE3) cells. Uba1 was purified with Ni-NTA, hydrophobic interaction (Hitrap Butyl HP, Cytiva), and gel filtration (Superdex 200 Increase 10/300, Cytiva) columns. Ubiquitin was purified with Ni-NTA, ion-exchange (Q-HP, Cytiva), and gel filtration (Superdex 200 increase 10/300) columns. To purify Rad6, the *RAD6* gene was inserted into vector pET28A (Novagen), and the plasmid was transformed into *E. coli* BL21 Rosetta (DE3) cells. Rad6 with an N-terminal 6× histidine tag was purified with Ni-NTA, ion-exchange (Q-HP), and gel filtration (Superdex 75 increase 10/300) columns, concentrated to 10 mg/ml in a buffer containing 20 mM TRIS (pH 7.5) and 200 mM sodium chloride, flash-frozen in liquid nitrogen, and stored at −80°C.

To purify the RNF-MD complex, the gene fragment encoding RNF20 residues 321–900 was inserted into vector pET28A together with an oligonucleotide encoding the strep and SUMO tags, and the gene fragment encoding RNF40 residues 327–926 was inserted into vector pMal-c2X (New England Biolabs). The recombinant RNF20 and RNF40 polypeptides contain strep-SUMO and maltose-binding-domain (MBP) tags, respectively. The plasmids were transformed separately into *E. coli* BL21 Rosetta (DE3) cells, which were induced with 0.25 mM IPTG for 16 h at 16°C. The RNF-MD complex was purified from the mixed cell extract with strep-tactin (Smart Life Sciences), dextrin (Smart Life Sciences), and gel filtration (Superdex 200 increase 10/300, Cytiva) columns, concentrated to 3 mg/ml in a solution containing 25 mM HEPES (pH 7.5) and 200 mM sodium chloride, flash-frozen in liquid nitrogen, and stored at −80°C.

To purify WAC-CT, the gene fragment encoding WAC residues 570–647 was inserted into vector pTXB1. The recombinant WAC-CT fused with the intein-CBD tag was purified with Ni-NTA, heparin, and gel filtration (Superdex 200 increase 10/300) columns. The protein was concentrated to 5 mg/ml in a solution containing 20 mM TRIS (pH 7.5) and 200 mM sodium chloride, flash-frozen in liquid nitrogen, and stored at −80°C.

Amino acid substitutions were introduced following the Quickchange (Agilent Technologies) protocol and verified by DNA sequencing. The substituted proteins were expressed and purified following the same protocols for the wild-type proteins.

Plasmids employed in this study are detailed in Supplementary Table S1.

### Crystallization and structure determination

Plate-like crystals of the Bre1-LR–Lge1-CT complex were obtained with the vapor diffusion sitting drop method at 4°C. The reservoir solution contains 120 mM ammonium citrate (pH 7.0), 12% polyethylene glycol (PEG) monomethyl ether (molecular weight 5000 Da), 0.5% β-mercaptoethanol, and 20 mM DTT. 25 mM hexamine cobalt chloride was supplemented to the complex solution before crystallization. The crystals were equilibrated in the reservoir solution supplemented with 25% (*v*/*v*) 2-methyl-2,4-pentanediol, flash cooled, and stored in liquid nitrogen before data collection. Crystals of the SeMet-substituted complex were obtained and treated with the same protocol.

A diffraction data set for the native Bre1-LR–Lge1-CT complex was collected at the Shanghai Synchrotron Radiation Facility (SSRF) beamline BL17U at 1.07 Å; another for the SeMet-substituted complex was collected on the National Facility for Protein Sciences (NFPS) beamline BL19U1 at SSRF at 0.98 Å. Diffraction data were indexed, integrated, and scaled with the XDS package [62]. Both the native and SeMet-substituted crystals belong to space group P2_1_ and their cell dimensions are similar (Table 1).

**Table 1.** Data collection and structure refinement statistics

	Native crystal	SeMet-substituted crystal
Data collection		
Space group	P2_1_	P2_1_
Cell dimensions		
*a, b, c* (Å)	114.97, 36.29, 170.77	112.69, 36.58, 171.23
α, β, γ (°)	90.00, 103.46, 90.00	90.00, 103.05, 90.00
Resolution (Å)	45.57–3.50 (3.59–3.50)	48.96–4.00 (4.10–4.00)
R_merge_	0.069 (2.221)	0.318 (2.305)
I / σI	9.85 (1.24)	9.77 (1.92)
CC_1/2_	0.999 (0.707)	0.999 (0.857)
Completeness (%)	96.5 (99.1)	99.8 (99.6)
Redundancy	6.5 (6.9)	31.3 (27.9)
		
Refinement		
Resolution (Å)	45.57–3.50 (3.72–3.50)	
No. reflections	17 191 (2 809)	
R_work_ / R_free_ (%)	28.79/32.50 (44.66/53.98)	
No. atoms		
Protein	6194	
Ligand/ion	0	
*B*-factors		
Protein	218.7	
Ligand/ion	−	
R.m.s. deviations		
Bond lengths (Å)	0.002	
Bond angles (°)	0.510	

Values in parentheses are for the highest-resolution shell.

The structure of the Bre1-LR–Lge1-CT complex was determined with molecular replacement with PHASER [63]. An analysis with COILS [64] indicated that the Bre1 LBD contains multiple coiled-coils. Ideal coiled-coil structures generated with CCFOLD [65] were used as search models. The first round of molecular replacement calculation identified two dimeric coiled-coils, which were fused to form one dimeric coiled-coil with 132 residues in each chain. Starting with this partial solution, the second round of molecular replacement calculation identified additional coiled-coil elements. Structure inspection and modification were carried out with COOT [66] and O [67], and structure refinement was carried out with PHENIX [68].

### Pulldown experiments

To investigate the Bre1–Lge1 interaction, 10 ml cells expressing intein-CBD-tagged Lge1-CT were lysed with sonication, and the cleared cell extract was incubated with chitin-agarose resin (New England Biolabs) in a binding buffer containing 20 mM TRIS (pH 7.5) and sodium chloride at the indicated concentrations at 4°C for 1 h. After washing the resin twice with the binding buffer, 0.15 mg Bre1-LR was applied to the resin. After another incubation at 4°C for 1 h, the resin was washed twice with the binding buffer, boiled in sodium dodecyl sulfate–polyacrylamide gel electrophoresis (SDS–PAGE) loading buffer, and analyzed by SDS–PAGE.

To investigate the interaction between RNF-MD and intein-WAC, 15 μg of intein-WAC was incubated with 10 μg of RNF-MD in a binding buffer containing 20 mM TRIS (pH 7.5) and 300 mM sodium chloride on ice for 15 min. The mixture was subsequently incubated with 20 μl chitin-agarose resin pre-equilibrated with the binding buffer for 1 h at 4°C. After washing the resin five times with the binding buffer, the resin was boiled in SDS–PAGE loading buffer and analyzed by SDS–PAGE.

### Isothermal titration calorimetry

ITC experiments were performed on a MicroCal PEAQ-ITC instrument (Malvern) at 25°C. Prior to the ITC experiments, Bre1-LR and Lge1-CT were exchanged in a buffer containing 20 mM TRIS (pH 7.5) and sodium chloride at the indicated concentrations. To characterize binding, Lge1-CT at concentrations of 50–60 μM was injected into a 300-μl cell that stores Bre1-LR at a concentration of 15 μM, 2 μl at a time. Data were analyzed with ORIGIN 7.0 (OriginLab).

### Surface plasmon resonance

Surface plasmon resonance (SPR) experiments were performed on a Biacore 8K instrument (Cytiva) at 25°C with a flow rate of 30 µl/min. To immobilize Lge1-CT or RNF-MD on a CM5 chip, these proteins were first diluted with HEPES buffer [20 mM HEPES (pH 7.5), 200 mM sodium chloride, 0.02% Tween 20] to 100 µg/ml and then with 10 mM sodium acetate (pH 4.5) to 1–3 µg/ml and flown over the chip for 60 s.

To characterize Bre1-LR binding to Lge1–CT, Bre1-LR was diluted with HEPES buffer to the indicated concentrations and flown over the chip immobilized with Lge1-CT for 120 s. Bre1-LR was subsequently dissociated from the chip by washing with HEPES buffer for 130 s.

To characterize intein-WAC binding to RNF-MD, intein-WAC was diluted with HEPES buffer to the indicated concentrations and flown over the chip immobilized with RNF-MD for 90 s. It was subsequently dissociated from the chip by washing with HEPES buffer for 100 s.

After each experiment, the chip was regenerated by flowing 10 mM glycine–HCl (pH 2.25) over the chip for 30 s. SPR data were analyzed with the Biacore Insight Evaluation software (Cytiva).

### *In vitro* nucleosome ubiquitination

The nucleosome was reconstituted following previously published methods with some modifications [53, 69]. Briefly, histone proteins H2A, H2B, H3, and H4 were co-expressed by inducing *E. coli* BL21 Rosetta (DE3) cells harboring a pETDuet-1 (Novagen) derived co-expression vector for these proteins with 0.5 mM IPTG for 2 h at 37°C. The recombinant H2B was fused with 6× histidine and FLAG tags. The nucleosome protein core complex was purified with Ni-NTA and gel filtration (Superdex 200 10/300) columns in a buffer containing 20 mM TRIS (pH 7.5) and 2 M sodium chloride. Double-stranded DNA containing the 147-bp Widom 601 sequence was produced by digesting a plasmid containing multiple copies of this sequence and purified with PEG (molecular weight 8000 Da) precipitation and ion-exchange chromatography (Q-HP). The nucleosome was reconstituted by incubating the nucleosome protein core complex with the double-stranded DNA at a molar ratio of 1:1 in a buffer containing 10 mM TRIS (pH 7.5), 2 M sodium chloride, and 1 mM ethylenediaminetetraacetic acid (EDTA) at 4°C, followed by dialysis that reduced the sodium chloride concentration to 250 mM overnight. The reconstituted nucleosome was purified with ion-exchange (Mono-Q, Cytiva) chromatography, dialyzed against a buffer containing 20 mM TRIS (pH 7.5), and concentrated to 2 mg/ml.

The nucleosome ubiquitination reaction mixture contains 50 mM TRIS (pH 8.0), 10 mM magnesium chloride, 45 mM imidazole, 0.1 mM DTT, 10 nM Uba1, 3 μM Rad6, 36 μM ubiquitin, 6 μM Bre1, 1 μM nucleosome, 3 mM ATP, and 4 μM Lge1, unless otherwise indicated. After incubating the mixture at 30°C for the indicated time, reactions were terminated by boiling in SDS–PAGE loading buffer and analyzed by western blotting with anti-FLAG (14793S, Cell Signaling Technology, RRID:AB_2572291, 1:3000 diluted) and horseradish peroxidase (HRP)-conjugated anti-rabbit (ZB-2301, ZSGB-BIO, RRID:AB_2747412, 1:5000 diluted) antibodies.

### Sedimentation experiment

A 5%–45% sucrose gradient of 12 ml was prepared in a centrifugation tube in a buffer containing 20 mM TRIS (pH 7.5), 10 mM potassium chloride, and 5 mM magnesium chloride. 200 micrograms of purified Bre1, Lge1, or their mixture were loaded onto the gradient. After centrifugation at 27 000 rpm for 15 h at 4°C with an SW41-Ti rotor (Beckman Coulter), 1 ml fractions were sequentially extracted from the top of the tube. Proteins in each fraction were precipitated by 10% trichloroacetic acid and collected by centrifugation. After washing with acetone, the resulting pellets were boiled in SDS–PAGE loading buffer and analyzed with SDS–PAGE.

### Yeast strains and plasmids

All the strains utilized in this study are derivatives of JKM139 [70], tGI354 [71], and MK203 [72]. The genotypes of the strains are detailed in Supplementary Table S2. Yeast strains were created by crossing with other genotypes or through one-step PCR cassette replacement, followed by transformation using standard procedures. The plasmids employed in this study are detailed in Supplementary Table S1.

### Yeast media and DNA damage sensitivity assays

All yeast strains employed in this study were cultured either on solid yeast media or in liquid YPD medium (2% peptone, 1% yeast extract, 2% glucose) at 30°C. For the DNA-damaging reagent sensitivity assays, 1:10 serial dilutions of overnight cultures of wild-type or mutant cells were spotted onto YPD plates with or without the indicated DNA-damaging reagents. The plates were incubated at 30°C for 2–4 days before imaging.

### Cellular H2B, Bre1, and Lge1 level analysis

Whole-cell yeast extracts were prepared using a trichloroacetic acid method as previously described [73]. Samples were analyzed by Western blotting to detect proteins of interest. Flag-tagged H2B, Bre1 fused with a 3× FLAG tag, Lge1 fused with a 3× HA tag, and GAPDH were detected with mouse anti-FLAG (F3165, Sigma–Aldrich, RRID: AB_259 529, 1:6000 diluted), anti-HA (M132-3, MBL, RRID:AB_10207271, 1:6000 diluted), and anti-GAPDH (AC033, ABclonal, RRID:AB_2769570, 1:50 000 diluted) antibodies, respectively, together with HRP-conjugated mouse IgG light chain binding protein (sc-516102, Santa Cruz Biotechnology, RRID: AB_2 687 626, 1:10 000 diluted).

### Co-immunoprecipitation

Yeast cells were cultured to the logarithmic phase, harvested by centrifugation, and lysed using a bead beater in a lysis buffer containing 100 mM HEPES (pH 8.0), 20 mM magnesium chloride, 150 mM sodium chloride, 10% glycerol, 0.4% nonidet *P*-40, and 0.1 mM EDTA. Protease and phosphatase inhibitors were supplemented to the lysis buffer. Benzonase (YEASEN) was added to each sample at a concentration of 500 U/ml prior to cell lysis. The lysate was clarified by centrifugation at 12 000 × *g* for 10 min at 4°C. The supernatant was incubated with an anti-FLAG (M185-3L, MBL, RRID:AB_11123930, 1:200 diluted) antibody at 4°C overnight with agitation, followed by incubation with protein G agarose beads for 4 h at 4°C. The beads were washed extensively with the lysis buffer at 4°C, and immunoprecipitated proteins were eluted by boiling the beads in SDS–PAGE loading buffer and detected with western blotting as described in the previous section.

### Analysis of DSB repair by ectopic recombination

Cells were cultured in YEPD medium to the logarithmic growth phase and serially diluted and plated onto YEPD or YEP-galactose plates, the latter of which induces HO expression. Colonies were enumerated after 3 days of incubation at 30°C. The survival rate was calculated by dividing the number of colonies formed on YEP-galactose plates by the number of colonies formed on YEPD plates and multiplying by the dilution factor.

### Chromatin immunoprecipitation

Chromatin immunoprecipitation (ChIP) experiments were carried out as previously described [73]. Briefly, exponentially growing yeast cells in YEPD medium (at a density of 1.2 × 10^7^ cells/ml) were harvested. Following chromatin shearing on a Diagenode Bioruptor, immunoprecipitations were carried out with anti-FLAG (14793S, Cell Signaling Technology, RRID: AB_2 572 291, 1:200 diluted) or anti-H2BUb1 antibodies (5546, Cell Signaling Technology, RRID: AB_10 693 452, 1:200 diluted). The purified DNA was analyzed with real-time qPCR with primers specific for the indicated genome loci with the following protocol: initial denaturation at 95°C for 10 min, followed by 40 cycles of denaturation (95°C for 15 s), primer annealing (60°C for 30 s), and elongation (72°C for 30 s).

### Chromatin fraction assay

Cells in log phase were exposed to 0.1% methyl methanesulfonate for 1 h, collected, washed with sterile water, and resuspended in 900 µl of ice-cold sorbitol solution [1 M sorbitol, 50 mM TRIS (pH 7.5)]. After digesting the cell walls with Zymolyase (Sigma) for 20 min at 37°C, the resulting spheroplasts were pelleted by centrifugation at 8 000 rpm for 2 min at 4°C, washed once with sorbitol solution, and resuspended in 400 µl lysis buffer [50 mM sodium chloride, 10 mM TRIS (pH 8.0), 5 mM magnesium chloride, 5 mM calcium chloride, 0.5 mM spermidine, 0.1% NP-40, 1 mM β-mercaptoethanol] supplemented with protease inhibitors. After incubation with rotation for 1 h at 4°C, one half of each sample was subjected to centrifugation at 12 000 rpm for 10 min at 4°C to separate the non-chromatin (soluble) and chromatin (pelleted) fractions. The remaining half was used to detect protein levels in the total cell extract. Protein levels were analyzed with Western blotting using antibodies for the FLAG tag (F3165, Sigma, RRID: AB_259 529, 1:3000 diluted), GAPDH (AC033, ABclonal, RRID: AB_2 769 570, 1:20 000 diluted), and histone H3 (A22348, ABclonal, RRID: AB_3 711 666, 1:6000 diluted).

### RNA sequencing

Yeast cells were initially plated on YEPD agar plates and incubated overnight, followed by subculturing in YEPD until reaching exponential growth. Cells were harvested promptly and stored at -80°C prior to total RNA extraction and library preparation. Library preparation and RNA sequencing were performed by Shanghai Majorbio Technology Co., Ltd.

### Reproducibility

Three independent experiments were conducted for the pulldown, Co-IP, ChIP, *in vitro* and *in vivo* H2BUb1 production, and ectopic recombination experiments.

### Statistics 

The average and standard deviations are shown for the quantification of experimental data presented in figures 3C, 4C-D, S13A, S14A and S15A, the p-values are derived from the two-tailed Student’s t-test.

## Results

### Structure of the Bre1–Lge1 complex

Our pulldown experiments confirmed the previously reported functions of Bre1 LBD and Lge1-CT in mediating the Bre1–Lge1 interaction (Supplementary Fig. S1A and B). To understand the structural basis of the interaction, we purified several Bre1–Lge1 complexes containing these regions and screened for their crystallization conditions. One complex containing Bre1 LBD and RING domains (Bre1-LR, Fig. 1A) and Lge1-CT crystallized. The crystals belong to space group P2_1_ and diffracted to a maximum resolution of 3.5 Å (Table 1). Bre1 LBD is predicted to contain several coiled-coils [53]. The structure was solved with molecular replacement using predicted coiled-coil structures as search models [65]. Model building at the relatively low resolution was facilitated by identifying the heptad repeating pattern of residues in the coiled-coils [64]. Bre1 residues 245–304 and 314–625 and Lge1 residues 268–331 were resolved in the electron density map. Electron densities for the Bre1 RING domain were not observed, suggesting that this domain is mobile in the crystal.

The structure revealed a 2:1 Bre1–Lge1 complex (Fig. 1B). The two Bre1 polypeptides in LBD form four dimeric coiled-coils (CC1–CC4) that constitute two strands. Strand 1 contains CC1-CC3 that are parallel to each other, strand 2 contains CC4 that is anti-parallel to CC1-CC3 and forms extensive interactions with them. Lge1-CT adopts a helical structure and binds in a cleft between strands 1 and 2. Overall, the complex adopts a highly elongated structure with a length of 270 Å and a maximum width of 40 Å. The flexibility that likely associates with such a structure may contribute to the relatively poor diffraction of the crystal.

To validate the structure, we produced crystals containing selenomethionine-substituted Bre1-LR and collected a data set at the selenium K-edge. The crystal diffracted to 4 Å (Table 1), and the anomalous signal contributed by the selenium atoms was significant up to 8 Å (Supplementary Fig. S2). Several strong peaks were found in the anomalous difference density map calculated with the anomalous signal, which are close to the three methionine residues in Bre1 LBD (Fig. 1C). These data indicated that the structure is correct.

### Structure prediction of the Bre1–Lge1 complex

We also carried out a structure prediction for the Bre1–Lge1 complex with AlphaFold2 [74]. The prediction suggests that the Bre1 domains are connected by flexible linkers. The predicted structures of RBD, LBD, and RING domains in Bre1 and Lge1-CT are homologous to the previously reported structures of Bre1 RBD [54, 55] and RING domain [75] and our crystal structure (Fig. 1D). Compared to the crystal structure, the positions of the two Bre1 polypeptides in LBD strand 1 are swapped in the predicted structure. Some global structural discrepancies are observed for Bre1 LBD between the predicted and crystal structures (Fig. 1E). Similar structural discrepancies have been reported for other AlphaFold-predicted structures [76]. Remarkably, the Bre1–Lge1 interface in the predicted and crystal structures are highly similar. Lge1-CT and its interacting regions in Bre1 in both structures can be aligned with a root mean square deviation (RMSD) of 1.68 Å for the Cα atoms (Fig. 1E). These regions are also associated with high predicted local distance difference test (pLDDT) scores in the predicted structure (Fig. 1F). Together, these data suggest that AlphaFold can accurately predict structures of the Bre1 domains and its interaction with Lge1 and the Bre1 structure could allow large domain movements during catalysis.

### Structure of the Bre1–Lge1 interface

The structure revealed extensive interfaces between Lge1-CT and both strands in Bre1 LBD (Supplementary Fig. S3A–E). The interface with strand 1 buries 1900 Å^2^ of surface area and is contributed by the middle (residues 287–306) and C-terminal (residues 307–332) regions in Lge1-CT, which interact with both Bre1 polypeptides and Bre1 polypeptide B, respectively. The interface with strand 2 buries 2700 Å^2^ of surface area and is primarily contributed by Bre1 polypeptide A, which mediates typical coiled-coil interactions with the entire Lge1-CT helix. Bre1 polypeptide B in strand 2 also mediates some interactions with the Lge1 C-terminus.

The Bre1–Lge1 interface contains many residues with hydrophobic side chains. Notably, the Lge1 C-terminus contains several such residues that are highly conserved among fungal Lge1 proteins, including Leu316, Ile318, Leu320, Leu325, Leu328, and Leu329 (Supplementary Fig. S4A). They interact with Bre1 residues K379’ (the ’ sign indicates residues in Bre1 polypeptide B), V382’, T386’, K486, Y487’, A489, A490, M491’, S496, and I497 (Supplementary Fig. S3D and E), most of which also contain hydrophobic side chains and are conserved among fungal Bre1 proteins (Supplementary Fig. S4B).

**Figure 2.**
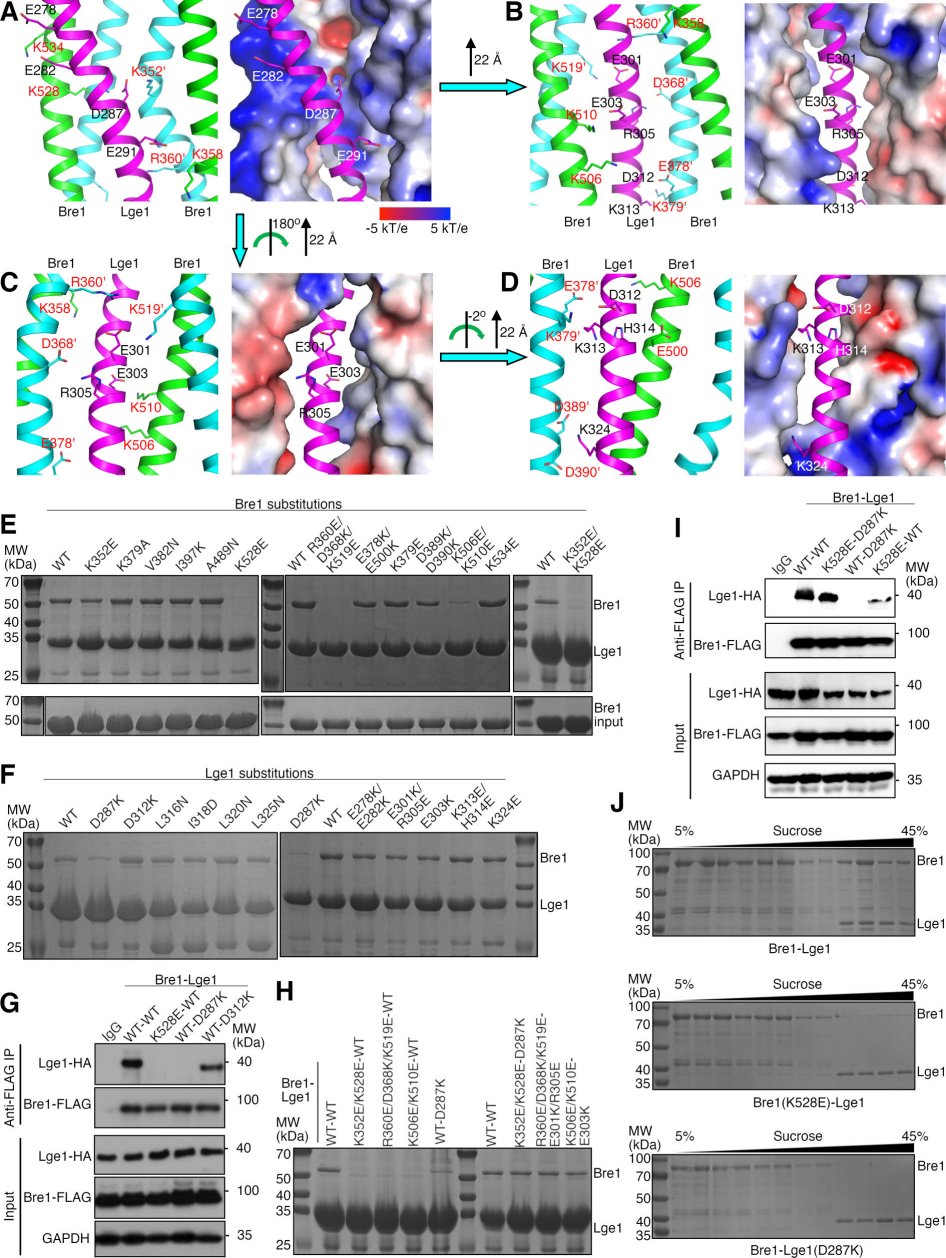
Structural insights into the Bre1–Lge1 interaction. (**A**–**D**) Electrostatic interactions at the Bre1–Lge1 interface. In the right panels, the surface of the Bre1 dimer is shown and colored according to the electrostatic potential. Bre1 and Lge1 residues are indicated by red and black/white labels, respectively. The ’ sign indicates residues in chain B of the Bre1 dimer. The transformation of the complex to produce the presented views is indicated. (E, F) Pulldown experiments probing the interaction between substituted Bre1 and Lge1 (**E**) and between Bre1 and substituted Lge1 (**F**). **(G)** Co-IP experiments probing the interaction between the wild-type and substituted Bre1 and Lge1. (**H**) Pulldown experiments probing effects of complementary charge-reversal substitutions on Bre1–Lge1 interaction. (**I**) Co-IP experiments probing effects of complementary charge-reversal substitutions on Bre1–Lge1 interaction in yeast cells. (**J**) Sedimentation of the Bre1–Lge1 mixture in a sucrose gradient. In panels (E), (F), (H), and (J), SDS–PAGE analysis of the pulldown or sedimentation experiments is presented. WT, wild-type.

The interface also contains many residues with charged side chains that mediate electrostatic interactions (Fig. 2A–D and Supplementary Fig. S3A–E). Such residues in Lge1 are distributed in 7 clusters throughout the entire helix, including Glu278 and Glu282 (cluster 1), Asp287 and Glu291 (cluster 2), Glu301 and Arg305 (cluster 3), Glu303 (cluster 4), Asp312 (cluster 5), Lys313 and His314 (cluster 6), and Lys324 (cluster 7). Bre1 residues interacting with these clusters include Lys534 (with cluster 1), Lys352’, Lys358, and Lys528 (with cluster 2), Arg360’, Asp368’, and Lys519’ (with cluster 3), Lys506 and Lys510 (with cluster 4), Lys379’ (with cluster 5), Glu378’ and Glu500 (with cluster 6), and Asp389’ and Asp390’ (with cluster 7). Remarkably, AlphaFold accurately predicted structures of these residue side chains. The only exceptions are Lys358 in Bre1 and Glu291 in Lge1 (Supplementary Fig. S3A–D).

### Key interactions at the Bre1–Lge1 interface

To probe the function of interactions at the Bre1–Lge1 interface, we introduced amino acid substitutions to disrupt these interactions. To disrupt the observed electrostatic interactions that usually play critical roles in the specific binding of protein partners [77], we introduced charge-reversal substitutions to the seven charged clusters in Lge1 and the Bre1 regions they interact with. Substitutions E278K/E282K, D287K, E301K/R305E, E303K, D312K, K313E/H314E, and K324E were introduced in Lge1, and K352E, K528E, K352E/K538E, R360E/D368K/K519E, E378K/E500K, K379E, D389K/D390K, K506E/K510E, and K534E were introduced in Bre1. We also introduced substitutions to disrupt the hydrophobic interactions mediated by the conserved Lge1 C-terminus. Substitutions L316N, I318D, L320N, and L325N were introduced in Lge1, and K379A, V382N, I397K, and A489N were introduced in Bre1.

We first carried out pulldown experiments with 300 mM sodium chloride to assess the effects of these substitutions. Consistent with the previous report [58], we found that Bre1-LR did not co-precipitate with the intein-CBD tag (Supplementary Fig. S1A) but strongly co-precipitated with the intein-CBD-fused Lge1-CT (Fig. 2E). We found that the co-precipitation was strongly inhibited by the K528E, K352E/K528E, R360E/D368K/K519E, or K506E/K510E substitutions in Bre1 (Fig. 2E) or the D287K substitution in Lge1 (Fig. 2F), whereas little effects were observed for the other substitutions.

To quantify the effects of the substitutions, we carried out ITC and SPR experiments (Supplementary Figs S6A and B and S7 and Table 2). Both experiments carried out with 200 mM sodium chloride revealed that Bre1-LR binds Lge1-CT with a *K*_D_ of ∼20 nM. In line with the pulldown experiments, neither experiment could detect significant binding between the K528-, K352E/K528E-, or R360E/D368K/K519E-substituted Bre1 and the wild-type Lge1. Similarly, the D287K substitution in Lge1 rendered the binding undetectable in the ITC experiment and caused an eight-fold increase in *K*_D_ in the SPR experiment. Contrary to the pulldown experiments, the ITC experiments indicated that the K506E/K510E substitution in Bre1 did not significantly alter the *K*_D._ To understand the effect of this substitution, we repeated the ITC (Supplementary Fig. S6B and Table 2) and pulldown (Supplementary Fig. S6C) experiments with different sodium chloride concentrations. These experiments revealed a salt concentration-dependent effect of this substitution. In both ITC and pulldown experiments, the substitution severely inhibited the Bre1–Lge1 interaction at high salt concentration (300 mM sodium chloride) but had little effect at a lower salt concentration (200 mM sodium chloride). However, this substitution rendered the binding undetectable in the SPR experiment carried out with 200 mM sodium chloride (Table 2 and Supplementary Fig. S7). The SPR experimental setup may have altered the salt concentration dependence.

**Table 2.** Summary of ITC and SPR experiments probing the Bre1–Lge1 interaction

		ITC experiments					SPR experiments		
Lge1	Bre1	N	ΔH (kcal/mol)	−TΔS (kcal/mol)	ΔG (kcal/mol)	*K*_D_ (nM)	*k*_a_ (1/Ms)	*k*_d_ (1/s)	*K*_D_ (nM)
Experiments with 200 mM sodium chloride									
WT	WT	0.396 ± 0.003	−50.5 ± 1.0	40.1	−10.40	24.5 ± 4.5	(6.45 ± 0.01) x 10^4^	(1.13 ± 0.002) x 10^−3^	17.5
WT	K352E	0.432 ± 0.011	−22.9 ± 1.3	13.7	−9.22	176 ± 45	(6.98 ± 0.13) x 10^5^	(5.35 ± 0.09) x 10^−2^	76.6
WT	K379A	0.415 ± 0.007	−44.1 ± 1.8	34.4	−9.67	82.3 ± 20.0	(5.50 ± 0.01) x 10^4^	(1.870 ± 0.004) x 10^−3^	34.0
WT	K379E	0.428 ± 0.008	−37.7 ± 1.6	27.8	−9.87	58.1 ± 19	(5.49 ± 0.01) x 10^4^	(2.270 ± 0.007) x 10^−3^	41.3
WT	V382N	0.378 ± 0.005	−53.1 ± 1.7	43.2	−9.84	61.2 ± 14.4	(9.53 ± 0.04) x 10^4^	(6.010 ± 0.008) x 10^−3^	63.0
WT	I397K	0.180 ± 0.005	−41.3 ± 2.1	31.9	−9.43	122 ± 42	(5.29 ± 0.03) x 10^4^	(2.160 ± 0.004) x 10^−3^	40.7
WT	A489N	0.375 ± 0.012	−52.1 ± 3.6	42.9	−9.30	154 ± 55	(5.81 ± 0.01) x 10^4^	(2.390 ± 0.004) x 10^−3^	41.1
WT	K528E	ND	ND	ND	ND	ND	ND	ND	ND
WT	K534E	0.469 ± 0.008	−33.9 ± 2.1	23.8	−10.2	36.9 ± 14.5	(5.68 ± 0.05) x 10^4^	(1.10 ± 0.01) x 10^−3^	19.4
WT	K352E/K528E						ND	ND	ND
WT	D389K/D390K	ND	ND	ND	ND	ND	(5.63 ± 0.01) x 10^4^	(4.250 ± 0.008) x 10^−3^	75.5
WT	E378K/E500K	0.256 ± 0.007	−40.7 ± 1.9	31.1	−9.56	98.0 ± 31.2	(1.98 ± 0.005) x 10^5^	(5.73 ± 0.01) x 10^−3^	29.0
WT	K506E/K510E	0.383 ± 0.005	−35.2 ± 0.9	25.1	−10.1	41.9 ± 10.1	ND	ND	ND
WT	R360E/D368K/K519E	ND	ND	ND	ND	ND	ND	ND	ND
D287K	WT	ND	ND	ND	ND	ND	(6.35 ± 0.06) x 10^4^	(9.21 ± 0.05) x 10^−3^	145
E303K	WT	0.587 ± 0.011	−46.7 ± 2.2	37.3	−9.39	132 ± 24	(3.780 ± 0.007) x 10^4^	(2.410 ± 0.004) x 10^−3^	63.8
D312K	WT	ND	ND	ND	ND	ND	(5.34 ± 0.01) x 10^4^	(1.380 ± 0.003) x 10^−3^	25.3
L316N	WT	0.293 ± 0.002	−65.4 ± 0.7	55.5	−9.94	52.2 ± 4.9	(8.40 ± 0.04) x 10^4^	(5.27 ± 0.01) x 10^−3^	62.8
I318D	WT	0.370 ± 0.022	−53.6 ± 0.8	43.2	−10.5	21.1 ± 3.7	(7.48 ± 0.02) x 10^4^	(1.550 ± 0.004) x 10^−3^	20.7
L320N	WT	0.370 ± 0.002	−49.9 ± 0.6	39.5	−10.4	23.7 ± 3.8	(8.20 ± 0.03) x 10^4^	(4.170 ± 0.007) x 10^−3^	50.8
K324E	WT	0.329 ± 0.001	−48.0 ± 0.7	37.0	−11	8.77 ± 2.09	(1.020 ± 0.002) x 10^5^	(5.09 ± 0.01) x 10^−3^	49.8
L325N	WT	0.367 ± 0.001	−61.2 ± 0.6	50.2	−11.0	8.06 ± 1.52	(5.59 ± 0.01) x 10^4^	(3.280 ± 0.003) x 10^−3^	58.7
E278K/E282K	WT	ND	ND	ND	ND	ND	(5.73 ± 0.02) x 10^4^	(5.48 ± 0.01) x 10^−3^	95.6
E301K/R305E	WT	0.585 ± 0.022	−19.4 ± 2.3	9.54	−9.86	59.7 ± 38.2	(5.28 ± 0.01) x 10^4^	(4.310 ± 0.009) x 10^−3^	81.7
K313E/H314E	WT	0.392 ± 0.005	−43.6 ± 1.1	33.4	−10.2	33.9 ± 8.4	(1.83 ± 0.05) x 10^5^	(9.47 ± 0.02) x 10^−3^	51.8
D287K	K352E/K528E						(1.66 ± 0.01) x 10^4^	(5.74 ± 0.01) x 10^−3^	346
E301K/E305K	R360E/D368K/K519E						(9.37 ± 0.03) x 10^3^	(5.43 ± 0.01) x 10^−3^	579
E303K	K506E/K510E						(2.89 ± 0.01) x 10^4^	(2.36 ± 0.01) x 10^−3^	81.7
Experiments with 300 mM sodium chloride									
WT	WT	0.318 ± 0.002	−38.8 ± 0.6	27.1	−11.7	2.7 ± 1.7			
WT	K506E/K510E	0.474 ± 0.060	−80.0 ± 55.5	72.9	−7.06	6720 ± 6580			

ND, not detectable.

The ITC and SPR experiments revealed effects of additional substitutions. Moderate inhibition of the Bre1–Lge1 interaction was observed for substitutions K352E, K379A, K379E, V382N, D389K/D390K, I397K, and A489N in Bre1, and L316N, E278K/E282K, E301K/R305E, E303K, and K313E/H314E in Lge1. They increased the *K*_D_ 2–7-fold. Little inhibition of the interaction was observed for substitutions E378K/E500K and K534E in Bre1 and D312K, I318D, L320N, L325N, and K324E in Lge1. Some of the substitutions, including D389K/D390K in Bre1 and D312K and E278K/E282K in Lge1, strongly reduced the binding signal in the ITC experiment (Supplementary Fig. S6A and Table 2) but not in the pulldown (Fig. 2E and F) or SPR experiments (Supplementary Fig. S7 and Table 2). They probably reduce the heat exchange with the environment in the binding process but have little effect on the binding affinity.

To assess the *in vivo* effects of the substitutions on the Bre1–Lge1 interaction, we carried out Co-IP experiments (Fig. 2G). In line with our pulldown, ITC, and SPR experiments, we found that Lge1 strongly co-precipitated with immunoprecipitated Bre1, and the co-precipitation was inhibited by the K528E substitution in Bre1 and the D287K substitution in Lge1, but not by the D312 substitution in Lge1. None of the substitutions significantly altered the Bre1 or Lge1 protein levels.

The above experiments revealed a set of critical electrostatic interactions, mediated by Asp287, Glu301, Glu303, and Arg305 in Lge1 and Lys352, Arg360, Asp368, Lys506, Lys510, Lys519, and Lys528 in Bre1. Consistently, we found that combined charge-reversal substitution on these residues, K352E/R360E/D368K/K506E/K510E/K519E/K528E in Bre1 (Bre1–7m) or D287K/E301K/E303K/R305E in Lge1 (Lge1–4m), severely inhibited the Bre1–Lge1 binding both *in vitro* (Supplementary Fig. S8A) and *in vivo* (Supplementary Fig. S8B). To probe the specificity of these interactions, we tested the effect of simultaneously introducing complementary charge-reversal substitutions at the Bre1 and Lge1 residues mediating these interactions. Complementary substitutions for K352E/K528E, R360E/D368K/K519E, and K506E/K510E in Bre1 and D287K in Lge1 that strongly inhibited the Bre1–Lge1 binding were tested. Remarkably, we found that all the complementary substitutions can restore the binding. Both our pulldown (Fig. 2H) and SPR (Table 2 and Supplementary Fig. S7) experiments indicated that the strongly inhibited binding by the Bre1 K352E/K528E substitution can be restored by the Lge1 D287K substitution, and vice versa. Similarly, the inhibited binding by Bre1 substitutions R360E/D368K/K519E and K506E/K510E can be restored by Lge1 substitutions E301K/R305K and E303K, respectively. A similar effect was observed for the Bre1–7m and Lge1–4m substitutions (Supplementary Fig. S8A). Consistent with the *in vitro* data, our Co-IP experiment indicated that the K528E-substituted Bre1 and the D287K-substituted Lge1 interact strongly *in vivo* (Fig. 2I). Restoration of the binding highlights the specificity of these electrostatic interactions. It also suggests that the substitutions do not introduce large structural changes, and the compromised binding is due to the loss of the electrostatic interactions. In support of this notion, we found that Bre1 substitutions K528E, K352E/K528E, R360E/D368K/K519E, and K506E/K510E did not alter its elution behavior in a gel filtration column (Supplementary Fig. S9A), and the K528E substitution did not affect the Bre1–Bre1 dimer interaction (Supplementary Fig. S9B).

Together, our data revealed a set of key electrostatic interactions critical for the Bre1–Lge1 binding and encodes the binding specificity. Our data also indicated that additional electrostatic interactions at the BreLge1 interface and hydrophobic interactions mediated by the conserved Lge1 C-terminus contribute to the Bre1–Lge1 binding.

### The Bre1–Lge1 interface is crucial for Bre1 recruitment to Lge1 condensates

Lge1 forms LLPS and recruits Bre1 to the resulting Lge1 condensates to stimulate the H2BUb1 catalysis [58]. To probe the role of the Bre1–Lge1 interface in this process, we tested whether substitutions that suppress the Bre1–Lge1 binding inhibit Bre1 recruitment to Lge1 condensates. In line with previous studies [58], we found that Lge1 sedimented to the high-molecular-weight region in a sucrose gradient (Supplementary Fig. S10A), whereas Bre1 alone sedimented to the low-molecular-weight region (Supplementary Fig. S10B). However, in the presence of Lge1, Bre1 co-sedimented with it to the high-molecular-weight region (Fig. 2J). The co-sedimentation is consistent with the recruitment of Bre1 to Lge1 condensates. Notably, we found little co-sedimentation in experiments with the K528E-substituted Bre1 or D287K-substituted Lge1, in which Bre1 and Lge1 still sedimented to the low- and high-molecular-weight regions, respectively (Fig. 2J). These data suggest that the Bre1–Lge1 interface is crucial for Bre1 recruitment to Lge1 condensates.

### The Bre1–Lge1 interface is crucial for the Bre1-catalyzed H2BUb1 reaction

**Figure 3.**
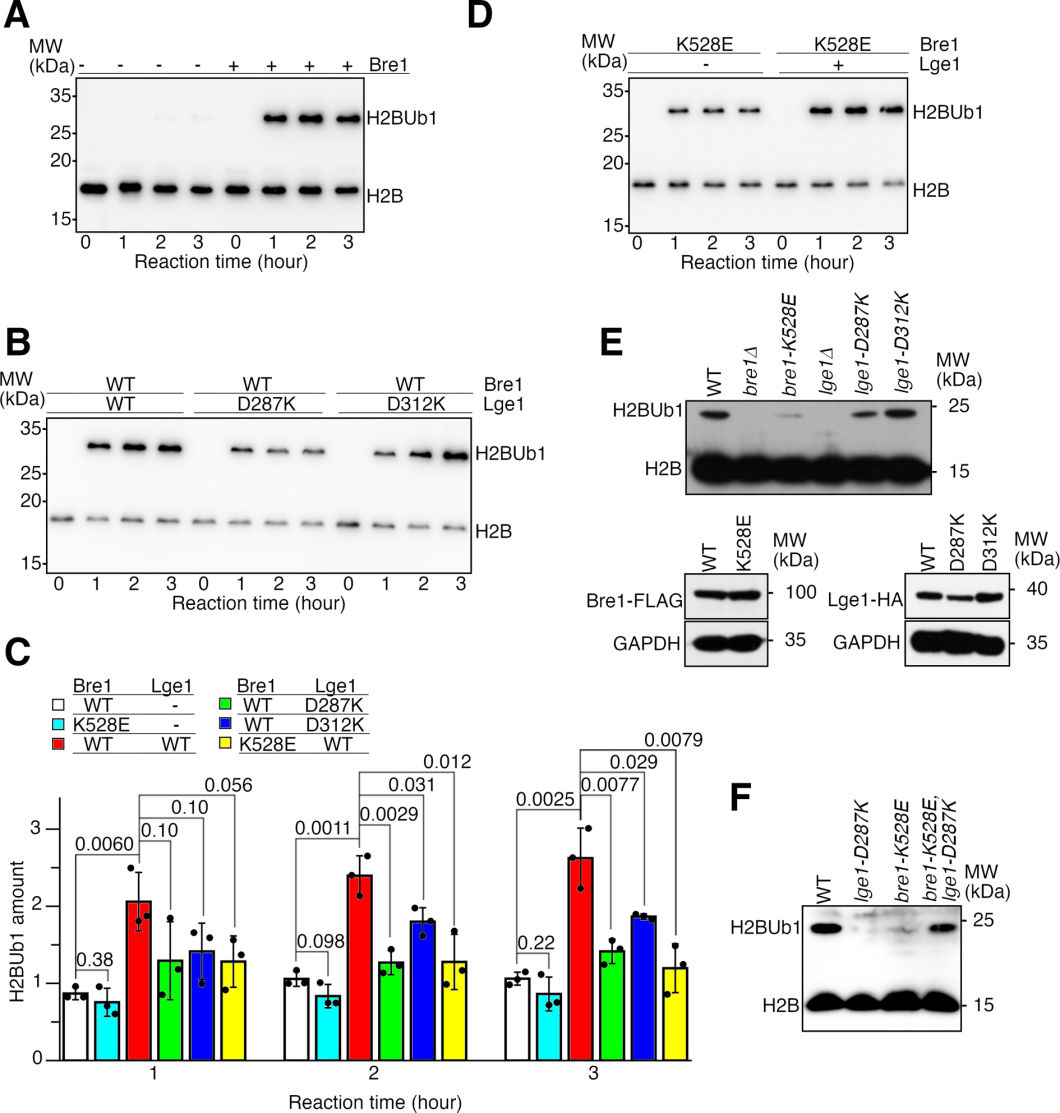
The Bre1–Lge1 interface is crucial for the H2BUb1 catalysis. (**A**) Bre1 catalyzes the H2BUb1 reaction *in vitro*. Western blot analysis of H2B is presented. (**B**) Effects of Lge1 substitutions on the H2BUb1 catalysis *in vitro*. (**C**) Quantification of the reactions. The H2BUb1 amount is calculated by dividing the intensity of the H2BUb1 band with the intensity of the H2B band at the beginning of the reaction. Band intensities were read with ImageJ (https://imagej.net). The average and standard deviation of three independent experiments (black dots) are presented. The *P*-values are derived from the two-tailed Student’s *t*-test. (**D**) Effects of the K532E substitution in Bre1 on the H2BUb1 catalysis *in vitro*. (**E**) Western blot probing the effects of Bre1 and Lge1 substitutions on the levels of H2BUb1, Bre1, and Lge1 *in vivo*. (**F**) Western blot probing the effects of complementary charge-reversal substitutions on H2BUb1 production *in vivo*.

To probe the function of the Bre1–Lge1 interface in the H2BUb1 reaction, we first assessed the effects of Lge1 and Bre1 substitutions with an *in vitro* ubiquitination assay with reconstituted nucleosome (Supplementary Fig. S11A and B). In line with previous reports [49, 53, 58], we found that Bre1 efficiently catalyzes the H2BUb1 reaction *in vitro* and the reaction is stimulated by Lge1 (Fig. 3A–C). We found that the stimulation was strongly and moderately reduced by the D287K and D312K substitutions in Lge1, respectively (Fig. 3B and C). A strong inhibition of the simulation was also observed for the K528E substitution in Bre1, which did not inhibit the H2BUb1 production in reactions catalyzed by Bre1 alone (Fig. 3C and D).

We next probed the effects of Bre1 and Lge1 substitutions on H2BUb1 production in budding yeast cells. In line with previous reports [42, 43, 56], we found that both Bre1 and Lge1 are required for efficient H2BUb1 production in budding yeast cells. Strong inhibition of the H2BUb1 production was observed for the K528E substitution in Bre1 and the D287K substitution in Lge1 (Fig. 3E), which can be restored by simultaneously introducing both substitutions (Fig. 3F). We also observed a stronger inhibition of the H2BUb1 production by the combined 7m or 4m substitutions in Bre1 or Lge1, respectively (Supplementary Fig. S11C), but not for the D312K substitution in Lge1 (Fig. 3E). None of the substitutions significantly altered the Bre1 or Lge1 protein levels (Fig. 3E and Supplementary Fig. S8B).

The effects of the substitutions on H2BUb1 production are well correlated with their effects on the Bre1–Lge1 interaction. Together, these data indicate that the Bre1–Lge1 interface plays a crucial role in the H2BUb1 catalysis.

### The Bre1–Lge1 interface is required for proper gene expression

Genome-wide studies in yeast and mammalian cells have demonstrated a positive correlation between transcriptional activity and H2BUb1 levels, and both increases and decreases in mRNA levels have been observed in cells lacking H2BUb1 [9]. To probe the function of the Bre1–Lge1 interface in transcription, we carried out RNA sequencing for the wild-type or the *lge1-D287K* or *bre1-K528E* mutant yeast cells. We found that the *lge1-D287K* mutation significantly altered the expression of 28 genes (5 upregulated and 23 downregulated; Supplementary Fig. S12A and Supplementary Table S3), and the *bre1-K528E* mutation significantly altered the expression of 218 genes (64 upregulated and 154 downregulated; Supplementary Fig. S12B and Supplementary Table S3). It is likely that cells have compensatory mechanisms that buffer against H2BUb1 loss, as suggested by a previous report that only a subset of genes was transcriptionally affected by RNF20 depletion and the lack of H2B ubiquitylation [78].

Next, we tested whether these mutations affect Bre1 recruitment to chromatin. In line with the previous report [56], our chromatin immunoprecipitation experiments indicated that deleting *LGE1* moderately reduced Bre1 recruitment to several actively transcribed genes (Supplementary Fig. S13A), whereas the *lge1-D287K* or *bre1-K528E* mutation did not cause noticeable reductions in Bre1 recruitment to these regions. Consistently, these mutations did not significantly alter the global association of Bre1 with chromatin (Supplementary Fig. S13B). However, the H2BUb1 level in these genes was significantly reduced by the *lge1-D287K* or *bre1-K528E* mutations (Supplementary Fig. S14). These data are in line with previous reports that Bre1 itself can interact with the nucleosome [49] and catalyze the H2BUb1 reaction *in vivo* [58]. They also suggest that disruption of the Bre1–Lge1 interaction abrogates the stimulation of Lge1 on Bre1’s catalytic activity rather than impair its loading on chromatin. Together, these results indicate that the interaction between Bre1 and Lge1 is necessary for optimal gene expression.

### The Bre1–Lge1 interface is important for the DNA damage responses and repair

**Figure 4.**
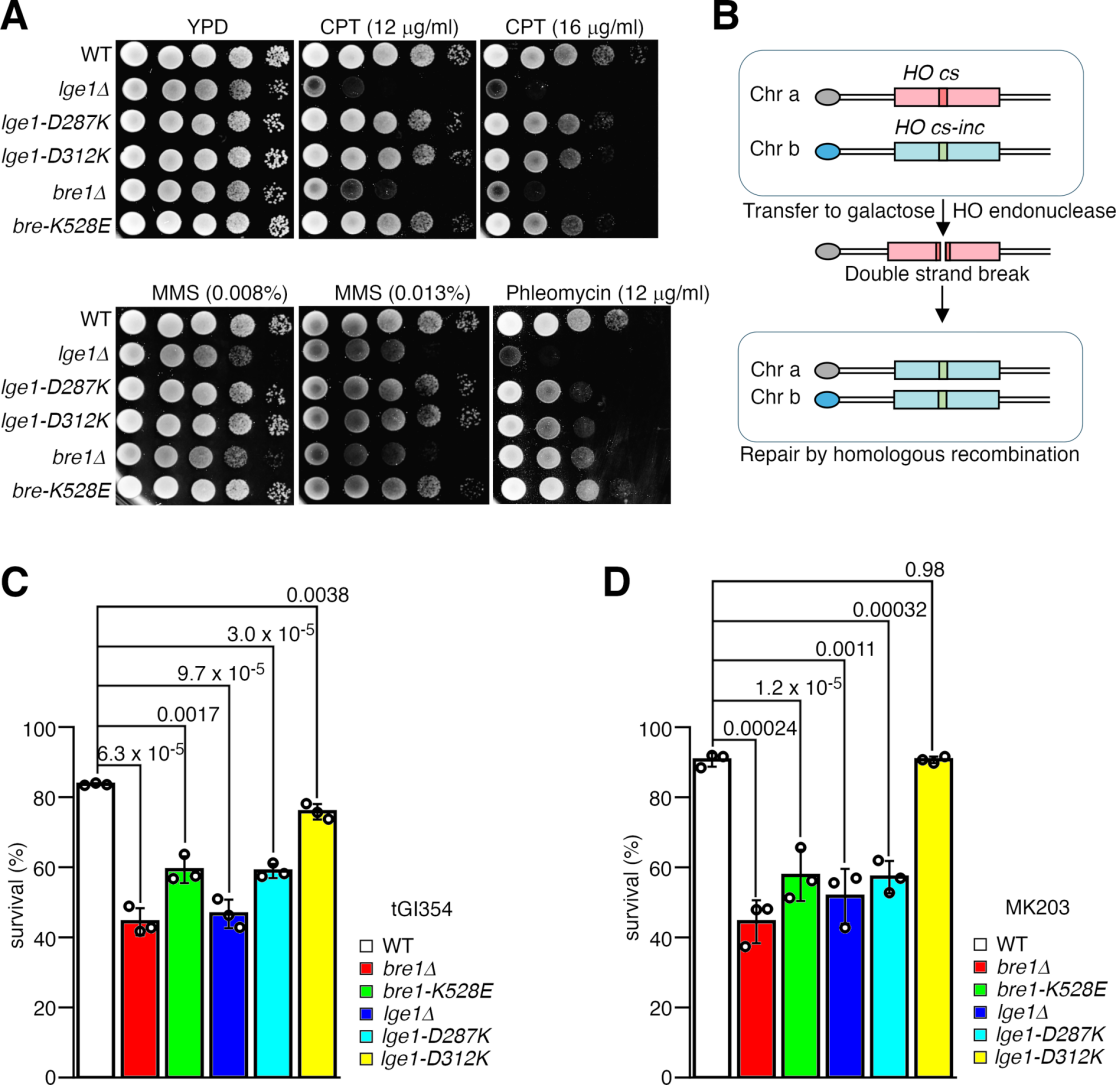
The Bre1–Lge1 interface is crucial for H2BUb1-regulated DNA damage responses and repair. (**A**) Effects of Bre1 or Lge1 knockout or their substitutions on cell survival in the presence of DNA-damaging agents. (**B**) Schematic of the ectopic recombination assays. HO cs, HO cleavage site; HO cs-inc, inactive HO cleavage site. (C, D) Results of the ectopic recombination experiments carried out with tGI354 **(C)** or MK203 cells **(D)**. The average and standard deviation of three independent experiments (hollow dots) are presented. The *P*-values are derived from the two-tailed Student’s *t*-test.

The H2BUb1 modification also plays an important role in promoting the DNA damage responses and repair including HR [31–35]. To assess the function of the Bre1–Lge1 interface in these processes, we first probed how substitutions in Bre1 or Lge1 affect cell survival in the presence of DNA-damaging agents (Fig. 4A). We found that the D287K and D312K substitutions in Lge1 and the K528E substitution in Bre1 reduced cell survival upon treatment with camptothecin or phleomycin, which can induce DSBs, but their effects were weaker than deleting the *BRE1* or *LGE1* genes. However, these substitutions did not render the cells sensitive toward methylmethane sulfonate, a DNA alkylation agent. We next probed the effects of Bre1 and Lge1 substitutions on HR repair using two independent ectopic recombination assays, in which cell survival reflecting HR repair of a single DSB created by the HO endonuclease was monitored (Fig. 4B) [71, 79]. These assays were carried out with the tGI354 or MK203 strains, in which the DSB was introduced into the *MATa* sequence on chromosome V or the *ura3* gene on chromosome V, and the HO-resistant donor template is located on chromosomes III or II, respectively. We found that in both systems, 80%–90% of the wild-type cells survived; the K528E substitution in Bre1 and the D287K substitution in Lge1 reduced the cell survival rate to less than 60%, but the D312K substitution in Lge1 did not have a significant effect. As a control, the *bre1Δ* or *lge1Δ* deletion mutant had a survival rate of 40%–50% (Fig. 4C and D). Notably, compared to the single K528E or D287K substitutions, the combined substitutions with all the key interface residues in Lge1 (Lge1–4m) or Bre1 (Bre1–7m) substituted impaired ectopic recombination repair to a greater extent and caused more pronounced sensitivity toward these DNA-damaging agents (Supplementary Fig. S15A and B). The effects of these substitutions on the DNA damage response and repair correlated well with their effects on the Bre1–Lge1 interaction and the H2BUb1 catalysis. Together, these data indicated that the Bre1–Lge1 interface is important for the H2BUb1-regulated DNA damage responses and HR repair.

### Structure prediction of the RNF20/RNF40 complex bound with WAC

**Figure 5.**
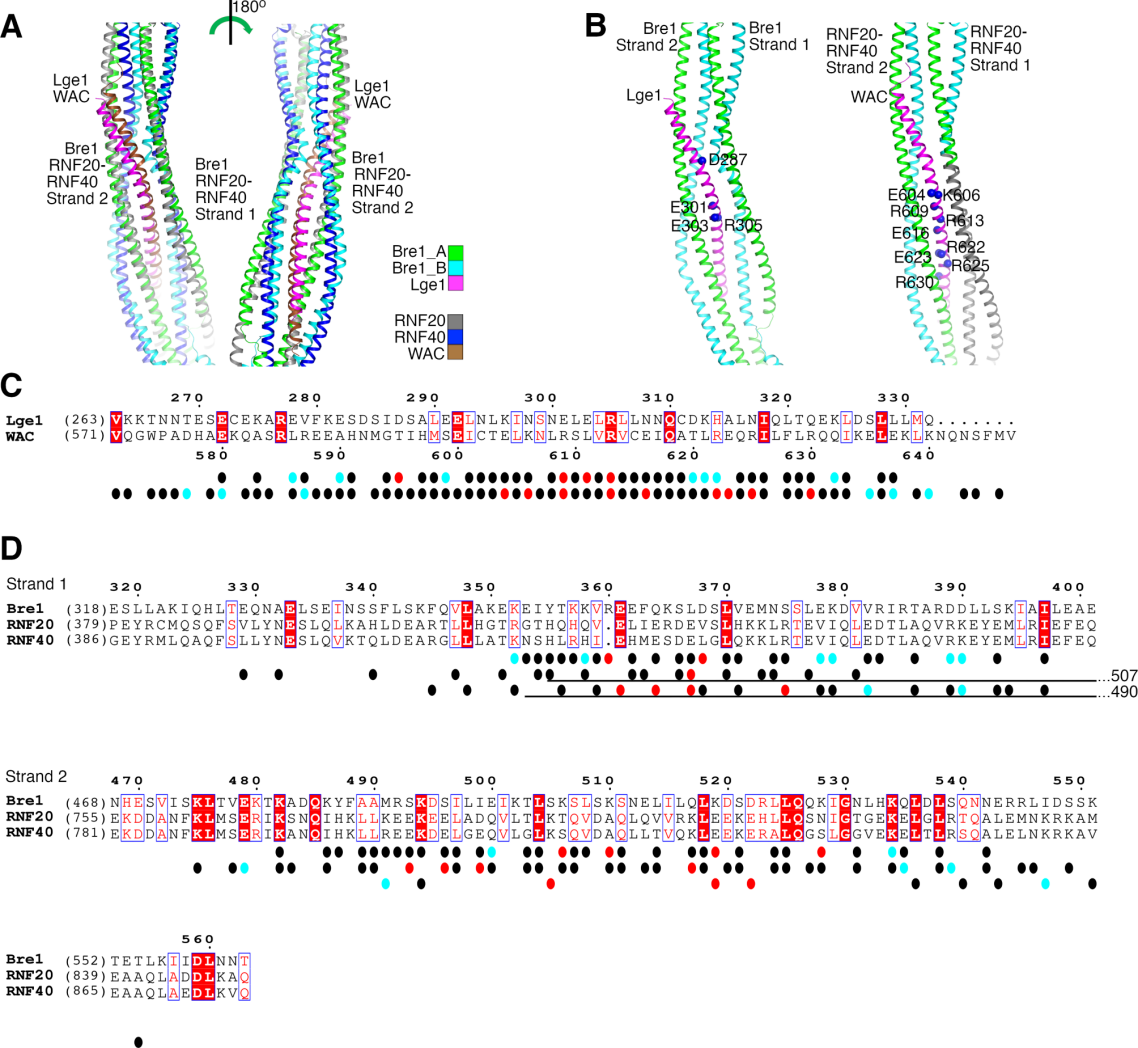
Comparison of the Bre1–Lge1 and RNF20/RNF40–WAC interfaces. (A, B) Structural comparison of Lge1-CT and WAC-CT and their binding regions in Bre1-LBD and RNF-MD, respectively. Crystal structure of the Bre1–Lge1 complex and the predicted structure of the RNF20/RNF40–WAC complex are superimposed (**A**) or presented side by side (**B**). In panel (B), residues mediating key electrostatic interactions in Lge1-CT and WAC-CT are indicated with blue spheres. The previously identified RNF20 and RNF40 regions crucial for WAC binding are highlighted in gray. (**C**) Sequence comparison of Lge1-CT and WAC-CT. Residue numbers above and below the sequences are for Lge1 and WAC, respectively. (**D**) Sequence comparison of Bre1, RNF20, and RNF40 regions mediating interactions with Lge1 or WAC. In panels (C) and (D), dots in black, red, and cyan indicate residues mediating hydrophobic, key, and additional electrostatic interactions. In panel (C), the dots in the first and second lines are for Lge1 and WAC, respectively. In panel (D), the dots in the first, second, and third lines are for Bre1, RNF20, and RNF40, respectively. The black lines in panel (D) indicate the previously identified RNF20 and RNF40 regions crucial for WAC binding.

To understand the interaction between the RNF20/RNF40 complex and WAC, we predicted a structure model of the RNF20/RNF40 complex bound with WAC-CT with AlphaFold2 [74]. We hypothesized that like the 2:1 Bre1–Lge1 complex, RNF20, RNF40, and WAC form a 1:1:1 complex. The predicted structure of such a complex (Supplementary Fig. S16A) suggests that RNF20 and RNF40 form a heterodimer consisting of N-terminal (NTD), middle (MD), and RING domains, with flexible linkers connecting NTD and MD. Like Bre1 LBD, MD in the RNF20/RNF40 complex (RNF-MD) is also predicted to adopt an elongated structure consisting of two strands, each containing a continuous or semi-continuous dimeric coiled-coil formed by RNF20 and RNF40 polypeptides. WAC-CT is predicted to adopt a helical structure and bind to a cleft between the two strands. The predicted structures of WAC-CT and its binding regions in RNF-MD are associated with high pLDDT scores (Supplementary Fig. S16B and C), suggesting highly reliable prediction [74]. The predicted structure of these regions is homologous to the structure of Lge1-CT and its binding regions in Bre1 LBD (Fig. 5A and B). After structure alignment, the RMSD of the Cα atoms in these regions in both structures is 4.29 Å. The sequence homology between these regions in the two complexes is very limited. The sequence identities between Lge1-CT and WAC-CT and their interacting regions in strands 1 and 2 in Bre1 LBD or RNF-MD are 10%, 8%, and 20%, respectively (Fig. 5C and D).

### Structure of the predicted RNF20/RNF40–WAC interface

**Figure 6.**
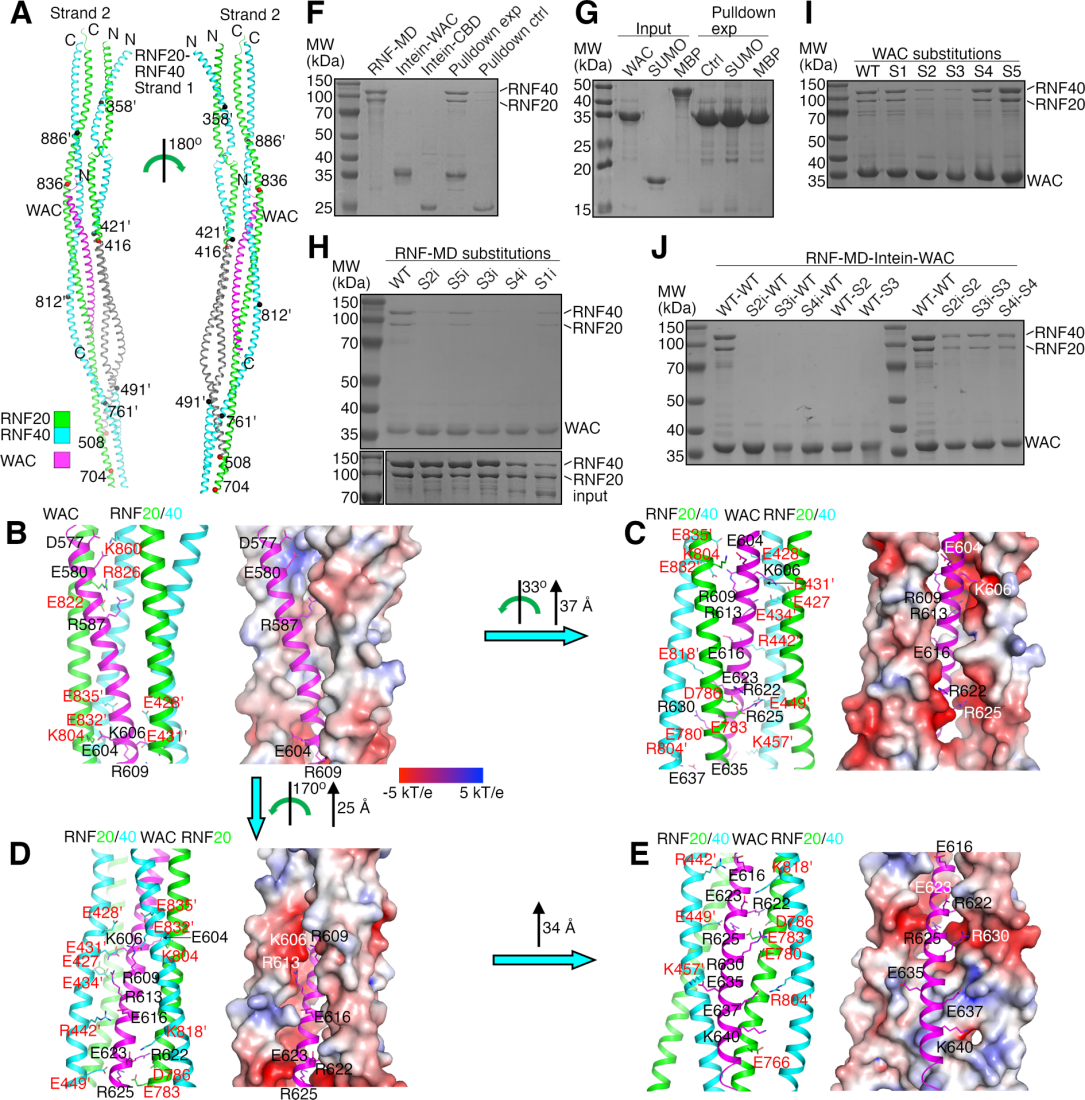
Structural insights into the RNF20/RNF40–WAC interaction. (**A**) AlphaFold predicted structure of the RNF20/RNF40 complex bound with WAC-CT. For clarity, only RNF-MD and WAC-CT regions are shown. RNF20, RNF40, and WAC are colored in green, cyan, and magenta, respectively. Previously identified regions in RNF20 and RNF40 crucial for the interaction with WAC are highlighted in gray. Boundaries of deletions made to identify these regions in RNF20 and RNF40 are indicated with red and black spheres, respectively. The coloring scheme is used throughout the paper unless otherwise indicated. (**B**–**E**) Electrostatic interactions at the predicted interface between the RNF20/RNF40 complex and WAC. In the right panels, the surface of the RNF20/RNF40 complex is shown and colored according to the electrostatic potential. Residues in RNF-MD and WAC are indicated with red and black/white labels, respectively. The ’ sign indicates RNF40 residues. The transformation of the complex to produce the presented views is indicated. (**F**) Pulldown experiments probing the interaction between intein-WAC and RNF-MD. In the control experiment (pulldown ctrl), intein-WAC is replaced by the intein-CBD tag. (**G**) Pulldown experiments probing the interaction between intein-WAC and the MBP or strep-SUMO tags. (H, I) Pulldown experiments probing the effects of substitutions in RNF-MD **(H)** or WAC **(I)** on the RNF20/RNF40–WAC interaction. (**J**) Pulldown experiments probing effects of complementary charge-reversal substitutions on RNF20/RNF40–WAC binding. In panels (F)–(J), SDS–PAGE analysis of the pulldown experiments is presented. Substitutions in WAC are D577K/E580K/R587E (S1), E604K/K606E/R609E/R613E/E616K (S2), R622E/E623K/R625E (S3), R630E (S4), and E635K/E637K/K640E (S5); in RNF-MD are E822K/R826E in RNF20 and K860E in RNF40 (S1i), E427K/K804E in RNF20 and E428K/E431K/E434K/R442E/E832K/E835K in RNF40 (S2i), E783K/D786K in RNF20 and E449K/K818E in RNF40 (S3i), D780K in RNF20 (S4i), E766K in RNF20 and K57E/R804E in RNF40 (S5i).

The predicted RNF20/RNF40–WAC interface is significantly more extensive than the Bre1–Lge1 interface (Fig. 6A). The interface between WAC and strand 1 in RNF-MD buries 3400 Å^2^ of surface area and is contributed by the entire WAC-CT helix, whose N-terminal, middle, and C-terminal regions interact with RNF20, both RNF20 and RNF40, and RNF40, respectively. The interface between WAC and strand 2 buries 5000 Å^2^ of surface area and is also contributed by the entire WAC-CT helix, which mediates typical coiled-coil interactions with RNF20 and additional interactions with RNF40.

The predicted interface contains many hydrophobic and electrostatic interactions (Supplementary Fig. S17A–E). The latter is mediated by 5 clusters of WAC residues with charged side chains and regions in RNF-MD carrying opposite charges (Fig. 6B–E). These WAC residues are distributed throughout the WAC-CT helix, including Asp577, Glu580, and Arg587 (cluster 1); Glu604, Lys606, Arg609, Arg613, and Glu616 (cluster 2); Arg622, Glu623, and Arg625 (cluster 3); Arg630 (cluster 4); and Glu635, Glu637, and Lys640 (cluster 5). Residues in RNF-MD they are predicted to interact with include Glu822 and Arg826 in RNF20 and Lys860 in RNF40 (with cluster 1); Glu427 and Lys804 in RNF20 and Glu428, Glu431, Glu434, Arg442, Glu832, and Glu835 in RNF40 (with cluster 2); Glu783 and Asp786 in RNF20 and Glu449 and Lys818 in RNF40 (with cluster 3); Glu780 in RNF20 (with cluster 4); and Glu766 in RNF20 and Lys457 and Arg804 in RNF40 (with cluster 5).

### Key interactions at the RNF20/RNF40–WAC interface

To probe the function of the interactions at the predicted RNF20/RNF40–WAC interface, we carried out pulldown experiments with purified WAC-CT and RNF-MD. The latter was purified sequentially with strep-tactin and dextrin resins, which bind to strep-SUMO-fused RNF20 and maltose-binding domain (MBP)-fused RNF40, respectively. Neither fusion polypeptide binds both resins, indicating that the purified RNF20 and RNF40 polypeptides constitute the heterodimeric RNF-MD (Supplementary Fig. S18). Consistent with the structure prediction, we found that RNF-MD co-precipitated with intein-CBD-tagged WAC-CT (intein-WAC) but not the intein-CBD tag (Fig. 6F), and the co-precipitation is not due to interactions between intein-WAC and the strep-SUMO or MBP tags (Fig. 6G).

We next introduced amino acid substitutions to disrupt the predicted interactions and assessed their effects with the pulldown experiment. We focused on residues mediating electrostatic interactions and introduced charge-reversal substitutions D577K/E580K/R587E (S1), E604K/K606E/R609E/R613E/E616K (S2), R622E/E623K/R625E (S3), R630E (S4), and E635K/E637K/K640E (S5) to clusters 1–5 of charged residues in WAC-CT, respectively. We also introduced charge-reversal substitutions in RNF-MD regions they are predicted to interact with. Substitutions E822K/R826E in RNF20 and K860E in RNF40 (S1i), E427K/K804E in RNF20 and E428K/E431K/E434K/R442E/E832K/E835K in RNF40 (S2i), E783K/D786K in RNF20 and E449K/K818E in RNF40 (S3i), D780K in RNF20 (S4i), and E766K in RNF20 and K57E/R804E in RNF40 (S5i) were introduced to residues predicted to interact with clusters 1–5, respectively. The pulldown experiment indicated that substitutions S2i, S3i, and S4i in RNF-MD and S2 and S3 in intein-WAC strongly inhibited the co-precipitation, whereas other substitutions had moderate or little effects (Fig. 6H and I).

To quantify the effects of the substitutions, we carried out SPR experiments (Supplementary Fig. S19 and Table 3). The SPR experiments indicated that intein-WAC binds to RNF-MD with a *K*_D_ of 504 nM. The substitutions S2i, S3i, and S4i in RNF-MD and S2 and S3 in intein-WAC inhibited the binding to undetectable levels, while the S4 substitution in intein-WAC increased the *K*_D_ two-fold. Other substitutions did not appear to have strong effects.

**Table 3.** Summary of SPR experiments probing the RNF20/RNF40–WAC interaction

WAC	RNF20/RNF40	*k*_a_ (1/Ms)	*k*_d_ (1/s)	*K*_D_ (nM)
WT	WT	(4.64 ± 0.11) x 10^4^	(2.34 ± 0.04) x 10^−2^	504
WT	S1i	(3.13 ± 0.03) x 10^4^	(1.68 ± 0.01) x 10^−2^	539
WT	S2i	ND	ND	ND
WT	S3i	ND	ND	ND
WT	S4i	ND	ND	ND
WT	S5i	(3.05 ± 0.05) x 10^4^	(1.42 ± 0.01) x 10^−2^	464
S1	WT	(3.20 ± 0.02) x 10^4^	(1.65 ± 0.01) x 10^−2^	514
S2	WT	ND	ND	ND
S3	WT	ND	ND	ND
S4	WT	(1.16 ± 0.02) x 10^4^	(1.38 ± 0.01) x 10^−2^	1190
S5	WT	(4.41 ± 0.06) x 10^4^	(1.43 ± 0.01) x 10^−2^	325
S2	S2i	(1.29 ± 0.003) x 10^4^	(3.38 ± 0.02) x 10^−3^	262
S3	S3i	(1.08 ± 0.003) x 10^4^	(3.08 ± 0.02) x 10^−3^	285
S4	S4i	(1.75 ± 0.02) x 10^4^	(4.35 ± 0.03) x 10^−2^	249

Substitutions in WAC are D577K/E580K/R587E (S1), E604K/K606E/R609E/R613E/E616K (S2), R622E/E623K/R625E (S3), R630E (S4), and E635K/E637K/K640E (S5); in RNF-MD are E822K/R826E in RNF20 and K860E in RNF40 (S1i), E427K/K804E in RNF20 and E428K/E431K/E434K/R442E/E832K/E835K in RNF40 (S2i), E783K/D786K in RNF20 and E449K/K818E in RNF40 (S3i), D780K in RNF20 (S4i), and E766K in RNF20 and K57E/R804E in RNF40 (S5i).

Both the pulldown and SPR experiments revealed strong inhibition of the RNF20/RNF40–WAC binding by charge-reversal substitutions at residues in charged clusters 2, 3, and 4 in WAC-CT or RNF-MD residues interacting with them. In line with the expected specificity of the electrostatic interactions mediated by these residues, both our pulldown (Fig. 6J) and SPR (Supplementary Fig. S19 and Table 3) experiments indicated that the strongly inhibited RNF20/RNF40–WAC binding by the S2 and S3 substitutions in WAC can be restored by substitutions S2i and S3i in RNF-MD, and vice versa, and by S4i in RNF-MD can be restored by S4 in WAC.

Together, the structure prediction and interaction experiments revealed a set of key electrostatic interactions at the RNF20/RNF40–WAC interface, mediated by WAC residues in clusters 2, 3, and 4, including Glu604, Lys606, Arg609, Arg613, Glu616, Arg622, Glu623, Arg625, and Arg630, and residues in RNF-MD they are predicted to interact with, Glu427, Glu780, Glu783, Asp786, and Lys804 in RNF20 and Glu428, Glu431, Glu434, Arg442, Glu449, Lys818, Glu832, and Glu835 in RNF40. These highly specific interactions are not only crucial for the RNF20/RNF40–WAC binding but also encode the binding specificity.

## Discussion

Lge1 is crucial for the Bre1 recruitment and stimulates the Bre1-catalyzed H2BUb1 reaction. Here, we provide a detailed picture of the Bre1–Lge1 interface, where we identified a network of key electrostatic interactions that are crucial for the Bre1–Lge1 binding and encode the binding specificity. The Lge1 residues mediating these interactions are clustered in the middle region of the Lge1-CT helix (Fig. 5B), suggesting that this region may act as an anchorage for the Bre1–Lge1 binding. Remarkably, two of the four Lge1 residues mediating the key interactions, Asp287 and Glu303, are strictly conserved among Lge1 proteins (Supplementary Fig. S4A). The Bre1 residues they interact with, Lys506, Lys510, and Lys528, are also highly conserved (Supplementary Fig. S4A and B). Such strong conservation is in line with their critical role in mediating the key Bre1–Lge1 interactions. We further found that disrupting the key interactions impaired the H2BUb1 catalysis and the H2BUb1-mediated transcription and DNA damage responses and repair. Our data provide insights into the mechanism of the Bre1–Lge1 interaction and highlight its importance in the H2BUb1 catalysis and its cellular function.

Like Lge1, WAC also binds to the RNF20/RNF40 complex to facilitate its recruitment and may stimulate the H2BUb1 reaction it catalyzes. We predicted the structure of the RNF20/RNF40–WAC complex with AlphaFold and found that the predicted RNF20/RNF40–WAC interface is homologous to the Bre1–Lge1 interface but much more extensive. The high pLDDT scores associated with the predicted interface suggest that the prediction is reliable. In support of this conclusion, the homologous Bre1–Lge1 interface predicted by AlphaFold is highly similar to our crystal structure and also associated with high pLDDT scores. Guided by the predicted structure, our interaction studies also revealed a network of key electrostatic interactions that are crucial for WAC binding to the RNF20/RNF40 complex and encode the binding specificity. These interactions are also mediated by a cluster of residues in the middle region of the WAC-CT helix and overlap with the Lge1 residue cluster mediating key interactions with Bre1 (Fig. 5B), including Arg613 that has an equivalent in Lge1 (Arg305, Fig. 5C). However, these interactions are mediated by a completely different set of residues in the RNF20/RNF40 complex and are entirely different from the key interactions at the Bre1–Lge1 interface (Fig. 5D). Together, the structure prediction and interaction studies suggest a model for the RNF20/RNF40–WAC interaction, which shares structure homology with the Bre1–Lge1 interaction but has a drastically different interacting mechanism.

A previous study reported that deleting residues 416–507 and 421–490 in RNF20 and RNF40, respectively, abolishes WAC binding to the RNF20/RNF40 complex [57]. Our structure prediction indicates that these regions mediate key electrostatic interactions between WAC and strand 1 in RNF-MD (Fig. 5B and D). The loss of binding is likely due to the loss of these key interactions. Several additional deletions made in that study are also expected to disrupt the WAC binding site (Fig. 6A) but did not abolish the binding. The retained binding may be contributed by interactions mediated by remaining regions in the WAC binding site. Together, this previous study supports our model and suggests that among the key electrostatic interactions we identified, those mediated by strand 1 in RNF-MD may play a more important role.

High levels of H2BUb1 may promote tumorigenesis in some cancers [38]. In addition, in the background of global H2BUb1 loss in several primary cancers including breast, lung, and colorectal cancers, H2BUb1 is selectively enriched at the coding regions of p53 target genes involved in DNA damage responses and genes involved in resistance to therapeutic drugs [39]. These findings suggest that suppressing H2BUb1 could be an effective treatment for multiple cancers. Our study shall facilitate future drug development targeting the RNF20/RNF40–WAC interface to attenuate the cellular H2BUb1 level.

In addition to mediating interactions with Lge1 or WAC, Bre1 LBD or RNF-MD also exerts a variety of other functions. Both the N-terminal Rad6-binding and C-terminal RING domains are expected to attach to the same side of Bre1-LBD or RNF-MD (Figs 1B and 6A). Such a structure could promote the coordination of these domains, which is crucial for the H2BUb1 reaction [53]. A recent study suggested that Bre1 LBD may contribute to the specificity of the Bre1-catalyzed H2BUb1 reaction [53]. Bre1 LBD or RNF-MD also mediates interactions with factors other than Lge1 or WAC. We have recently found that Bre1 and the RNF20/RNF40 complex interact with RPA to facilitate their recruitment to replication forks, DNA breaks [80], or mitotic centromeres [81]. Moreover, we have shown that Bre1 or the RNF20/RNF40 complex interacts with Rad51 and Srs2 or FBH1 to regulate the Rad51-single-strand DNA filament formation during HR [82]. Interestingly, the identified binding sites for RPA, Rad51, and Srs2 in Bre1 overlap with the Lge1 binding site, and the RPA binding site in the RNF20/RNF40 complex overlaps with the WAC binding site. Our study provides a starting point to study these important functions of Bre1 LBD and RNF-MD.

## Supplementary Material

gkaf1514_Supplemental_Files

## Data Availability

Diffraction data and the refined structure of the Bre1–Lge1 complex have been deposited into the protein data bank (www.rcsb.org), with the accession code 9M3I. RNA sequencing data have been deposited into the Sequence Read Archive (https://www.ncbi.nlm.nih.gov/sra/) with the accession code PRJNA1327771.

## Notes

Present address: Jiangsu Key Laboratory of New Drug Research and Clinical Pharmacy, Xuzhou Medical University,209 Tongshan Road, Xuzhou 221004, Jiangsu,P.R. China
