# Supplementary material for: Structural insights into the Bre1–Lge1 and RNF20/RNF40–WAC interactions critical for H2B ubiquitination
Source: Nucleic Acids Res. 2026 Jan 14;54(2):gkaf1514. doi: 10.1093/nar/gkaf1514 (PMC12802937; doi:10.1093/nar/gkaf1514)
Supplement: gkaf1514_Supplemental_Files [file gkaf1514_supplemental_files.zip › SI_r2.pdf]

Table S1 Plasmids used in this study

Plasmid name	Recombinant DNA	Source
pWX1	*pRS316-Lge1-3×HA*	This study
pWX2	*pRS316-lge1-D287K-3×HA*	This study
pWX3	*pRS316-lge1-D312K-3×HA*	This study
pBre1-FL	*pET28A-Bre1-FL*	This study
pBre1-LR	*pET28A-Bre1-LR*	This study
pBre1-RBD	*pET28A-Bre1-RBD*	This study
pBre1-LBD	*pET28A-Bre1-LBD*	This study
pLge1-FL	*pTXB1-Lge1-FL*	This study
pLge1-CT	*pTXB1-Lge1-CT*	This study
pLge1-dCT	*pTXB1-Lge1-dCT*	This study
pLge1-dYR	*pTXB1-Lge1-dYR*	This study
pLge1-His-FL	*pET28A-Lge1-FL*	This study
pRNF20-MD	*pET28A-strep-SUMO-RNF20-MD*	This study
pRNF40-MD	*pMAL-C2X-MBP-RNF40-MD*	This study
pWAC-CT	*pTXB1-WAC-CT*	This study
pHist	*pETDute-1-H2A-FLAG-H2B-H3-H4*	This study
pRad6	*pET28A-Rad6*	This study
pUba1	*pET28A-Uba1*	(1)

Table S2 Strains used in this study

Strain name	Parental strain	Genotype	Source
JKM139		*MATa ho hml::ADE1 hmr::ADE1 ade1-100 leu2-3,112 trp1::hisG lys5 ura3-52 ade3::GAL::HO*	(2)
tGI354		*MATa-inc arg5,6::MATa-HPH ade3::GAL::HO hmr::ADE1 hml::ADE1 ura3-52*	(3)
MK203		*MATa-inc ura3::HOcs (V) lys2::ura3-HOcs inc (1.2kb) ade3::GALHO leu2-3,112 his3-11,15 trp1-1 ade2-1 can1-100*	(4)
yXJ545	JKM139	*URA3-FLAG-HTB1*	This study
yXJ513	JKM139	*lge1-D287K-TRP1 KanMX-FLAG-HTB1*	This study
yXJ514	JKM139	*lge1-D312K-TRP1 KanMX-FLAG-HTB1*	This study
yXJ516	JKM139	*bre1-K528E-TRP1 KanMX-FLAG-HTB1*	This study
yXJ549	JKM139	*lge1::KanMX URA3-FLAG-HTB1*	This study
yGL060	JKM139	*bre1::TRP1 KanMX-FLAG-HTB1*	This study
yXJ517	tGI354	*lge1-D287K-TRP1*	This study
yXJ518	tGI354	*lge1-D312K-TRP1*	This study
yXJ520	tGI354	*bre1-K528E-KanMX*	This study
yXJ526	tGI354	*lge1::KanMX*	This study
yDL025	tGI354	*bre1::KanMX*	This study
yXJ521	JKM139	*lge1-D287K-TRP1*	This study
yXJ522	JKM139	*lge1-D312K-TRP1*	This study
yXJ524	JKM139	*bre1-K528E-KanMX*	This study
yXJ527	JKM139	*lge1::KanMX*	This study
yDL020	JKM139	*bre1::KanMX*	This study
yXJ530	MK203	*lge1::KanMX*	This study
yXJ531	MK203	*lge1-D287K-TRP1*	This study
yXJ532	MK203	*lge1-D312K-TRP1*	This study
yXJ533	MK203	*bre1::KanMX*	This study
yXJ534	MK203	*bre1-K528E-KanMX*	This study
yZL016	JKM139	*Bre1-3×FLAG-KanMX*	This study
yXJ525	JKM139	*bre1-K528E-3×FLAG-KanMX*	This study
yXJ546	JKM139	*pRS316-Lge1-3×HA Bre1-3×FLAG-KanMX*	This study
yXJ547	JKM139	*pRS316-lge1-D287K-3×HA Bre1-3×FLAG-KanMX*	This study
yXJ548	JKM139	*pRS316-lge1-D312K-3×HA Bre1-3×FLAG-KanMX*	This study
yXJ559	JKM139	*pRS316-Lge1-3×HA bre1-K528E-3×FLAG-KanMX*	This study
yXJ585	JKM139	*pRS316-lge1-D287K-3×HA bre1-K528E-3×FLAG-KanMX*	This study
yXJ584	JKM139	*pRS316-lge1-D287KE301KE303KR305E-3×HA Bre1-3×FLAG-KanMX*	This study
yXJ583	JKM139	*pRS316-Lge1-3×HA bre1-K352ER360ED368KK506E K510EK519EK528E -3×FLAG-KanMX*	This study
yXJ570	JKM139	*Bre1-3×FLAG-KanMX lge1-D287K-TRP1*	This study

yXJ571	JKM139	*Bre1-3×FLAG-KanMX lge1-D312K-TRP1*	This study
yXJ572	JKM139	*Bre1-3×FLAG-URA3 lge1::KanMX*	This study
yXJ580	JKM139	*bre1-K352ER360ED368KK506EK510EK519EK528E-TRP1 URA3-FLAG-HTB1*	This study
yXJ578	JKM139	*lge1-D287KE301KE303KR305E-TRP1 URA3-FLAG-HTB1*	This study
yXJ573	JKM139	*pRS316-Bre1-3×HA Bre1-3×FLAG-KanMX*	This study
yXJ574	JKM139	*pRS316-Bre1-3×HA bre1-K528E-3×FLAG-KanMX*	This study
yXJ576	MK203	*lge1-D287KE301KE303KR305E-TRP1*	This study
yXJ577	MK203	*bre1-K352ER360ED368KK506EK510EK519EK528E-TRP1*	This study
yXJ586	JKM139	*lge1-D287K-TRP1 bre1-K528E-KanMX HphMX-FLAG-HTB1*	This study

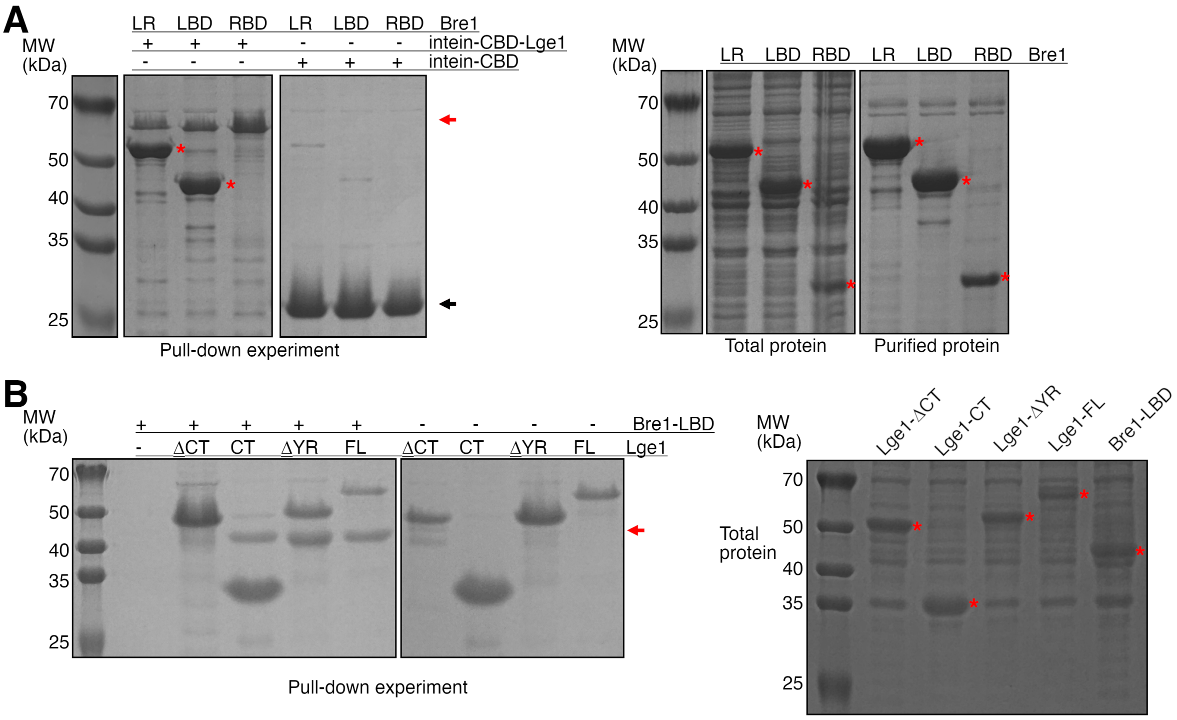

Figure S1 SDS-PAGE analysis of pulldown experiments probing the interaction between Bre1 and Lge1 and their fragments. (A) Pulldown experiments probing the interaction between Bre1 fragments and Lge1. Analysis of intein-CBD-Lge1 or the intein-CBD tag together with the indicated Bre1 fragments precipitated with the chitin resin is presented. In the right panel, SDS-PAGE analysis of the related cell extracts and Ni-NTA purified Bre1 fragments from them are presented. The red and black arrows indicate the intein-CBD-Lge1 fusion protein and the intein-CBD tag, respectively; the red stars indicate the Bre1 fragments. (B) Pulldown experiments probing the interaction between Bre1-LBD and Lge1 fragments. Analysis of the indicated intein-CBD fused Lge1 fragments precipitated with the chitin resin in the presence or absence of Bre1-LBD is presented. In the right panel, SDS-PAGE analysis of the related cell extracts is presented. The red arrow indicates Bre1-LBD, the red stars indicate Lge1 fragments and Bre1-LBD in the cell extract.

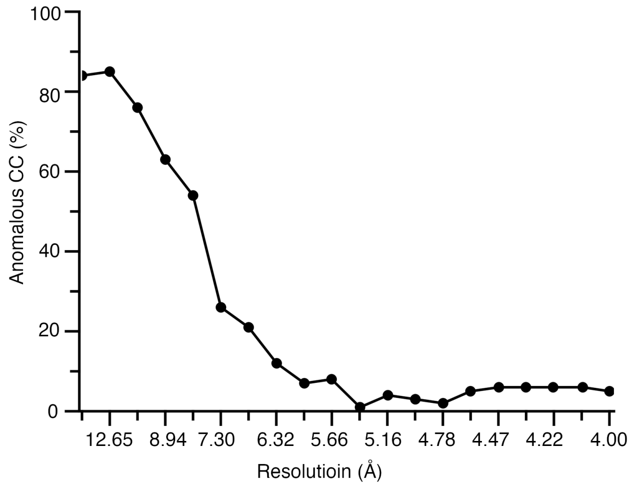

Figure S2 Anomalous signal in the data set collected on a SeMet-substituted crystal. Anomalous CC, anomalous correlation.

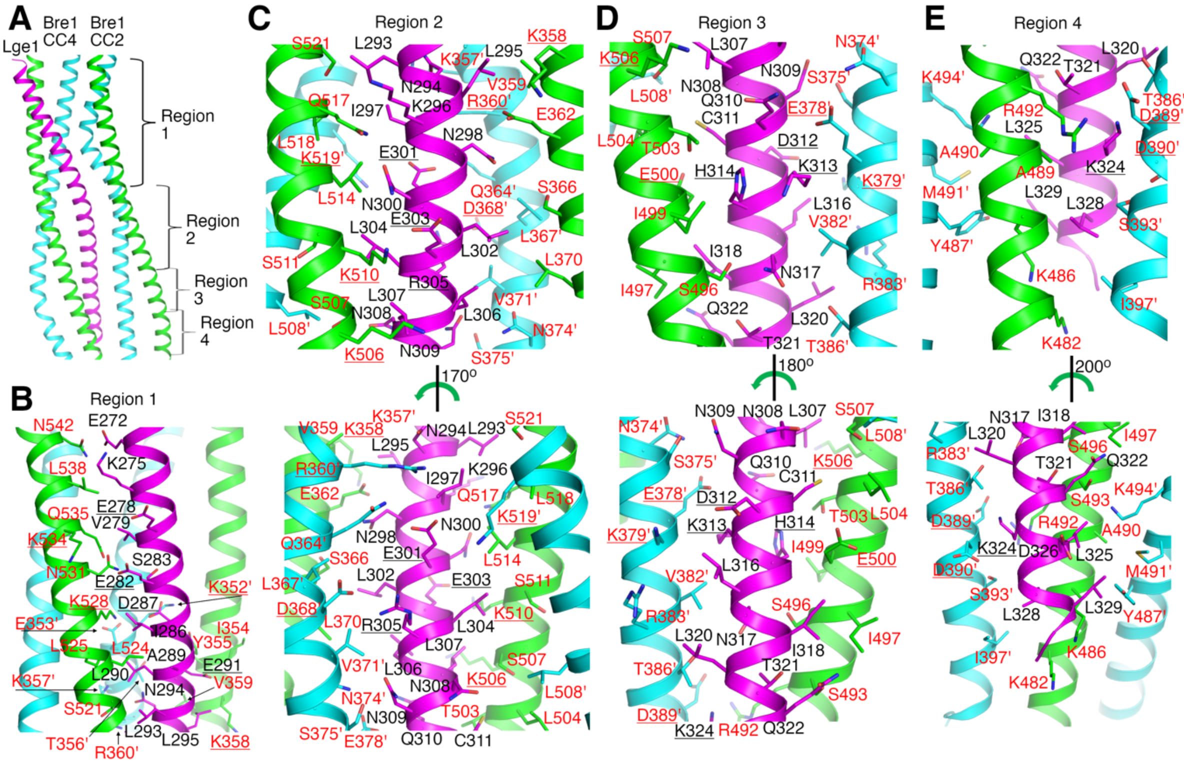

Figure S3 Interactions at the Bre1-Lge1 interface. The interface is divided into regions 1-4 (A) and interactions mediated by these regions are shown in panels (B)-(E). Bre1 and Lge1 residues are labeled in red and black, respectively. The ’ sign indicates residues in chain B of the Bre1 dimer. Residues mediating electrostatic interactions are indicated by underlined labels.

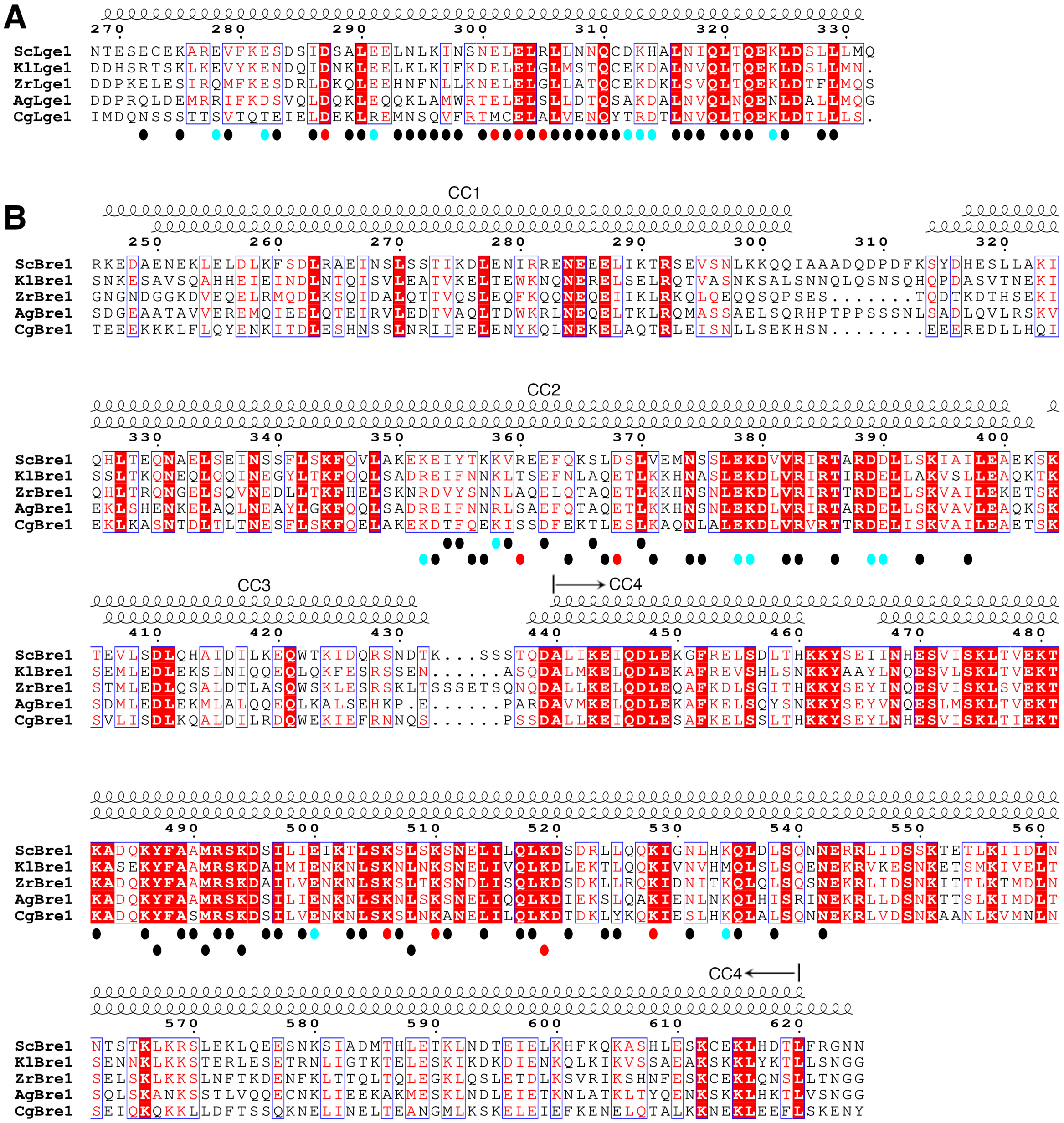

Figure S4 Sequence alignment of fungal Lge1 (A) and Bre1 (B) proteins. The Lge1-CT and Bre1 LBD regions are shown. Dots in black, red and cyan indicate residues mediating hydrophobic, key and additional electrostatic interactions, respectively. Dots in the first and second lines in panel (B) are for chains A and B in the Bre1 dimer, respectively. Secondary structural elements for the budding yeast Bre1 and Lge1 are indicated above the sequences. Secondary structural elements in lines 1 and 2 in panel (B) are for chains A and B in the Bre1 dimer, respectively. Sc, *Saccharomyces cerevisiae*; Kl, *Kluyveromyces lactis*; Zr, *Zygosaccharomyces rouxii*; Ag, *Ashbya gossypii*; Cg, *Candida glabrata*.

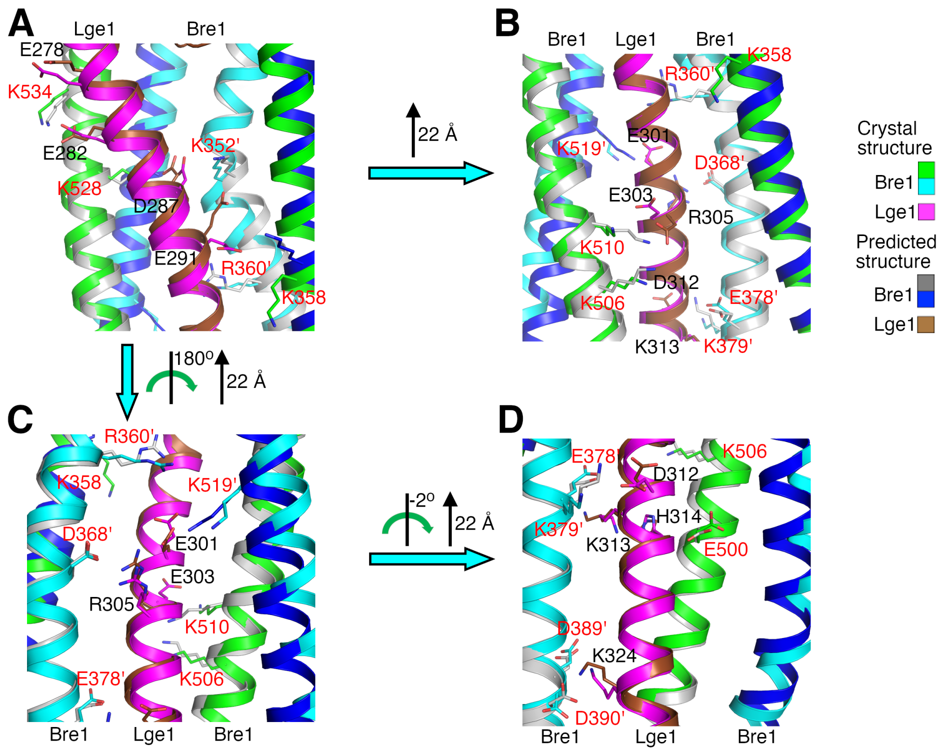

Figure S5 Comparison of the crystal and Alphafold-predicted structures of the Bre1-Lge1 complex. The Bre1-Lge1 interface region is presented. Residues mediating electrostatic interactions are shown. Views in panels (A)-(D) are identical to views in Figs 2A-D.

A

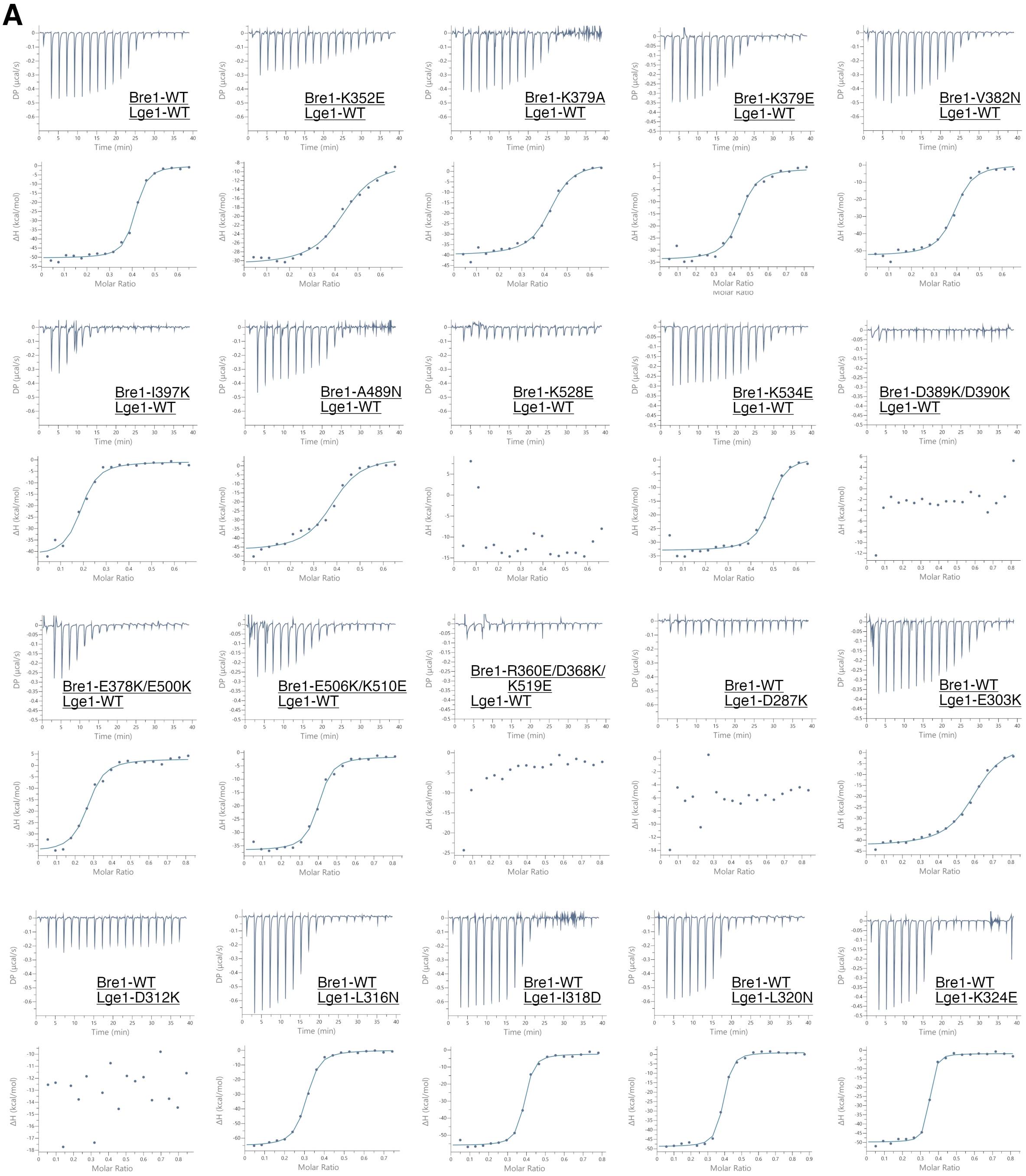

Continued on the next page

Continued from the previous page

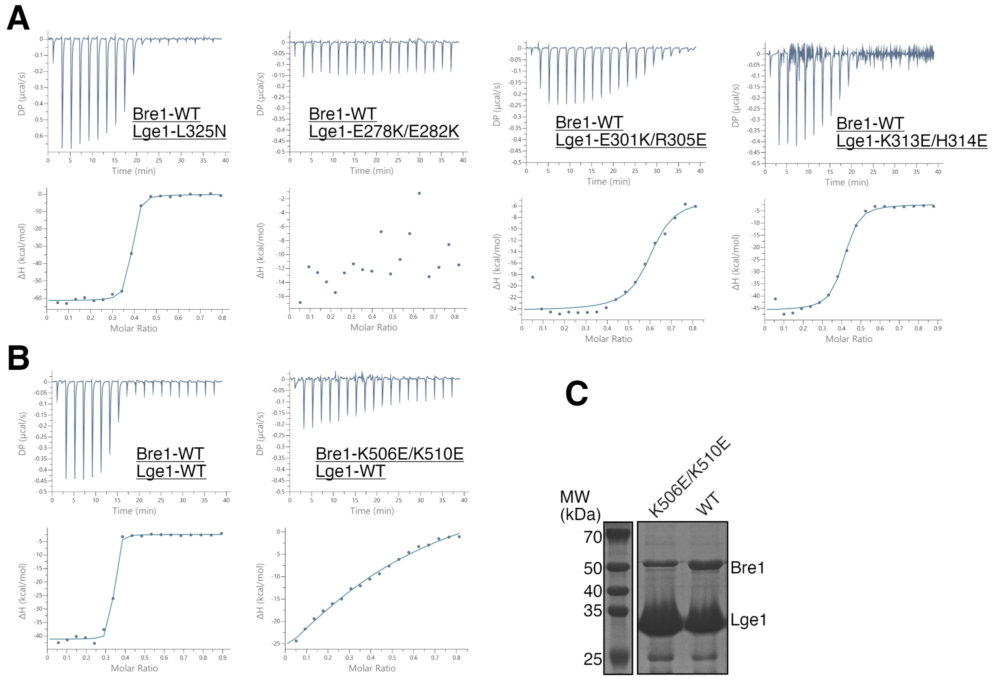

Figure S6 ITC and pulldown experiments probing interactions at the Bre1-Lge1 interface. (A)-(B) ITC experiments carried out with 200 mM (A) and 300 mM (B) sodium chloride. (C) Pulldown experiments carried out with 200 mM sodium chloride. Experiments with the wild-type and K506E/K510E-substituted Bre1 are presented. WT, wild-type.

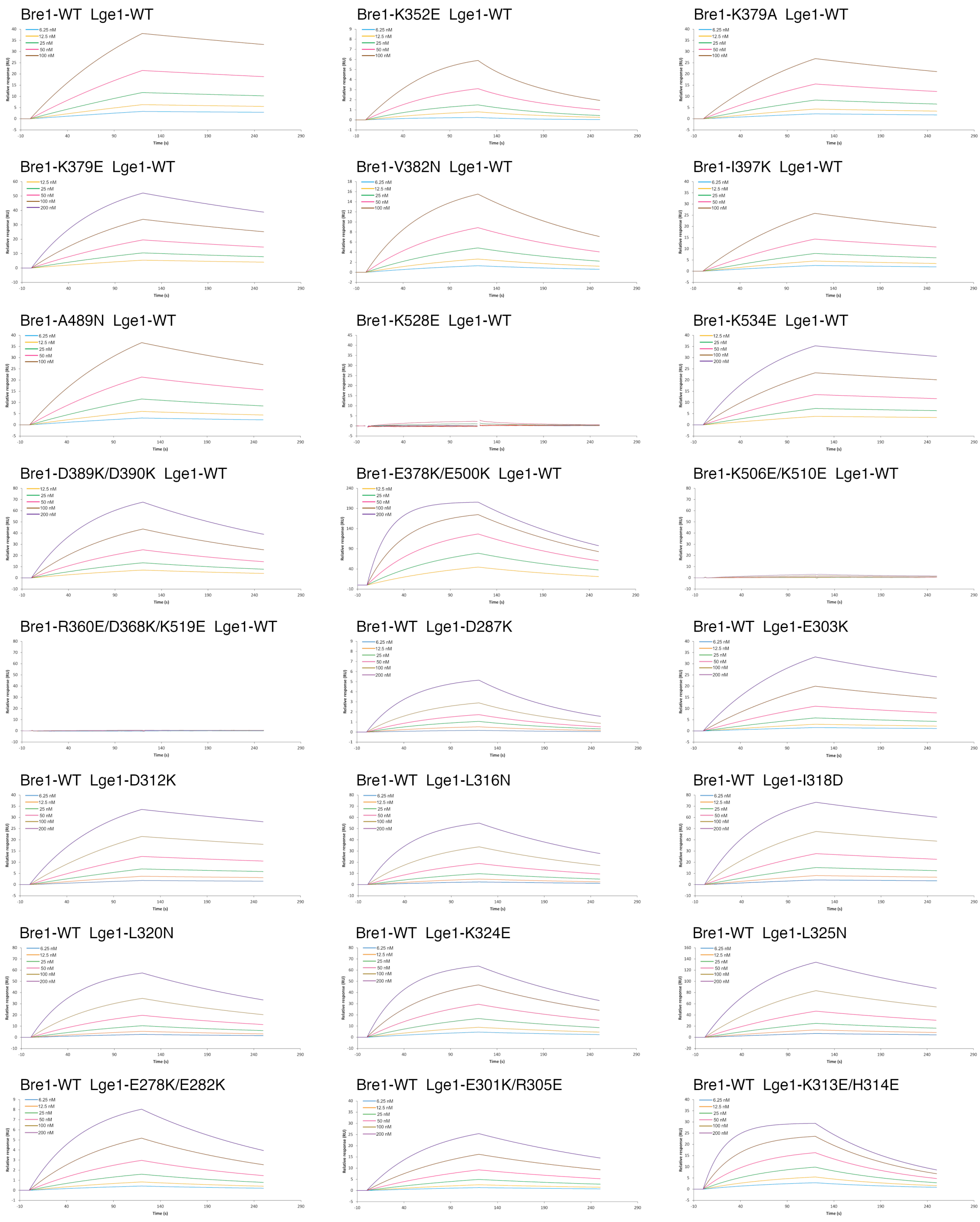

Continued on the next page

Continued from the previous page

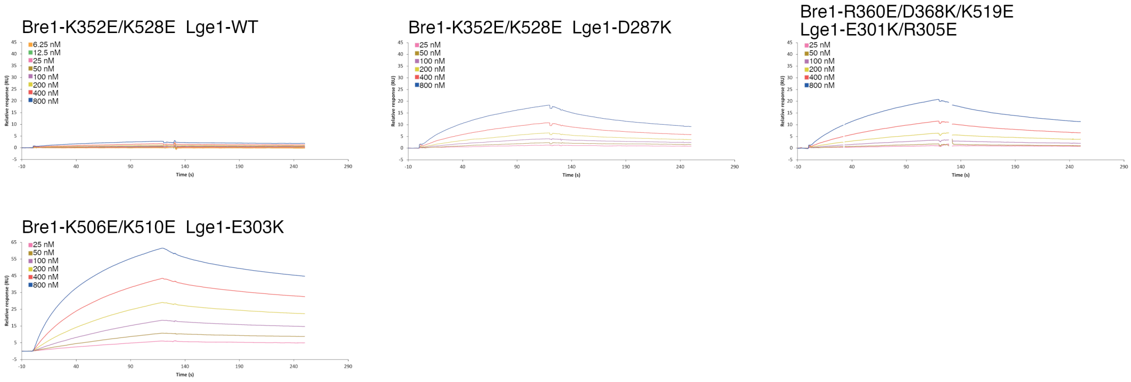

Figure S7 SPR experiments probing interactions at the Bre1-Lge1 interface.

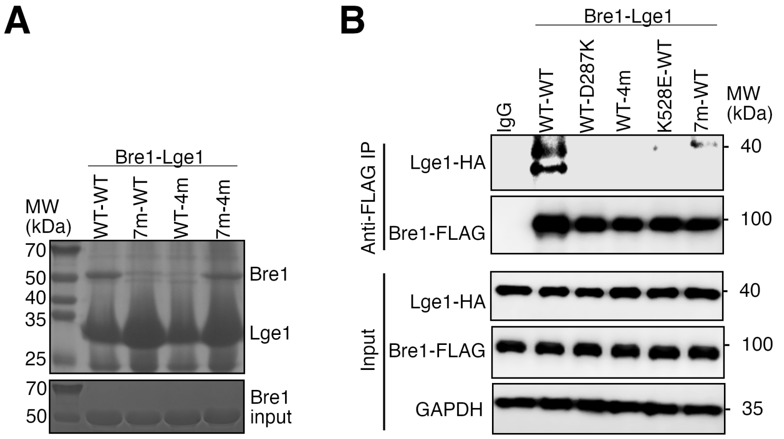

Figure S8 Effects of combined charge-reversal substitutions on the Bre1-Lge1 interaction. (A) Pulldown experiments probing the effects of the combined charge-reversal substitutions. (B) Co-IP experiments probing the effects of the combined charge-reversal substitutions. 7m, the K352E/R360E/D368K/K506E/K510E/K519E/K528E substitution in Bre1; 4m, the D287K/E301K/E303K/R305E substitution in Lge1.

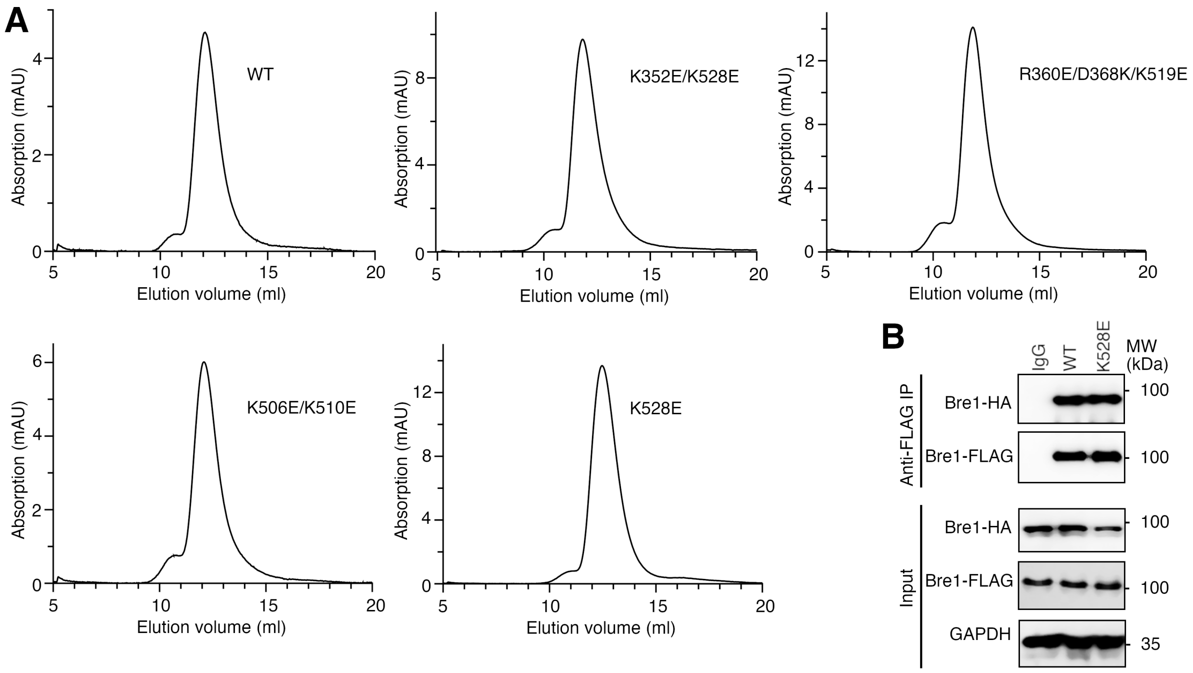

Figure S9 Substitutions in Bre1 do not alter its global structure or its dimerization. (A) Gel filtration characterization of wild-type and substituted Bre1-LR fragments. The proteins were characterized with a Superdex 200 10/300 column (Cytiva) in a buffer containing 20 mM TRIS (pH8.0) and 200 mM sodium chloride. (B) Co-IP experiments probing the Bre1-Bre1 dimer interaction for the wild type and K528E-substituted Bre1.

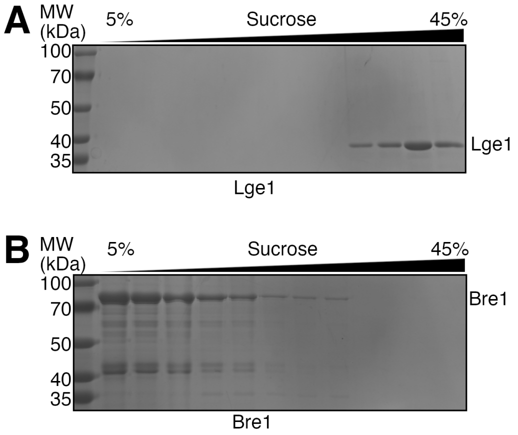

Figure S10 Sedimentation of Bre1 (A) or Lge1 (B) in a sucrose gradient. SDS-PAGE analysis of the fractions is presented.

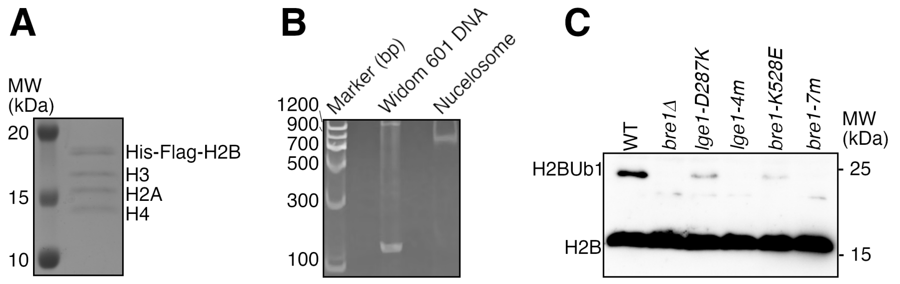

Figure S11 The Bre1-catalyzed H2BUb1 reaction. (A) SDS-PAGE analysis of the histone octamer. (B) Native PAGE analysis for the free and nucleosome-incorporated Widom 601 DNA. (C) Effects of the combined mutations on H2BUb1 production *in vivo*. *Bre1-7m*, the K352E/R360E/D368K/K506E/K510E/K519E/K528E mutation in *bre1*; *lge1-4m*, the D287K/E301K/E303K/R305E mutation in *lge1*.

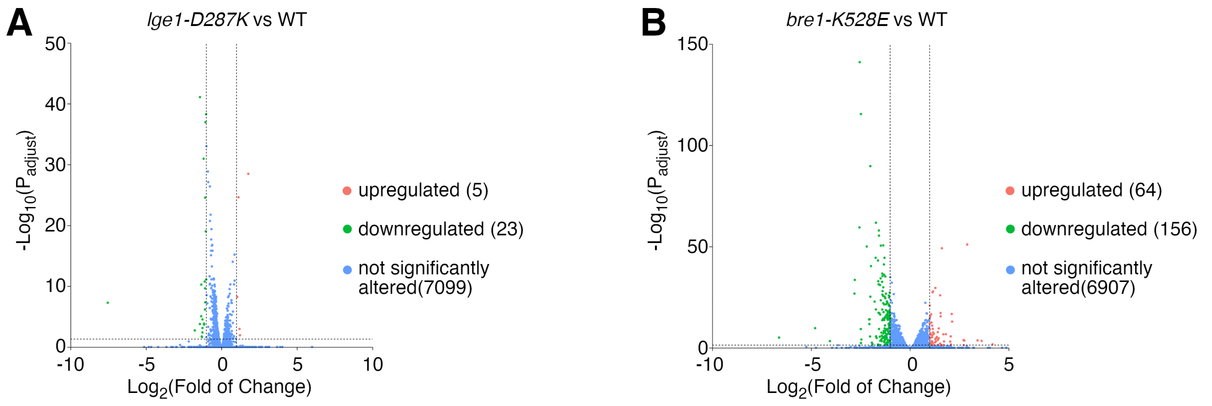

Figure S12 The Bre1-Lge1 interface is required for proper gene expression. (A) Comparison of RNA-sequencing results of the wild-type budding yeast cells and cells carrying the *lge1-D287K* mutation. (B) Comparison of RNA-sequencing results of the wild-type budding yeast cells and cells carrying the *bre1-K528E* mutation.

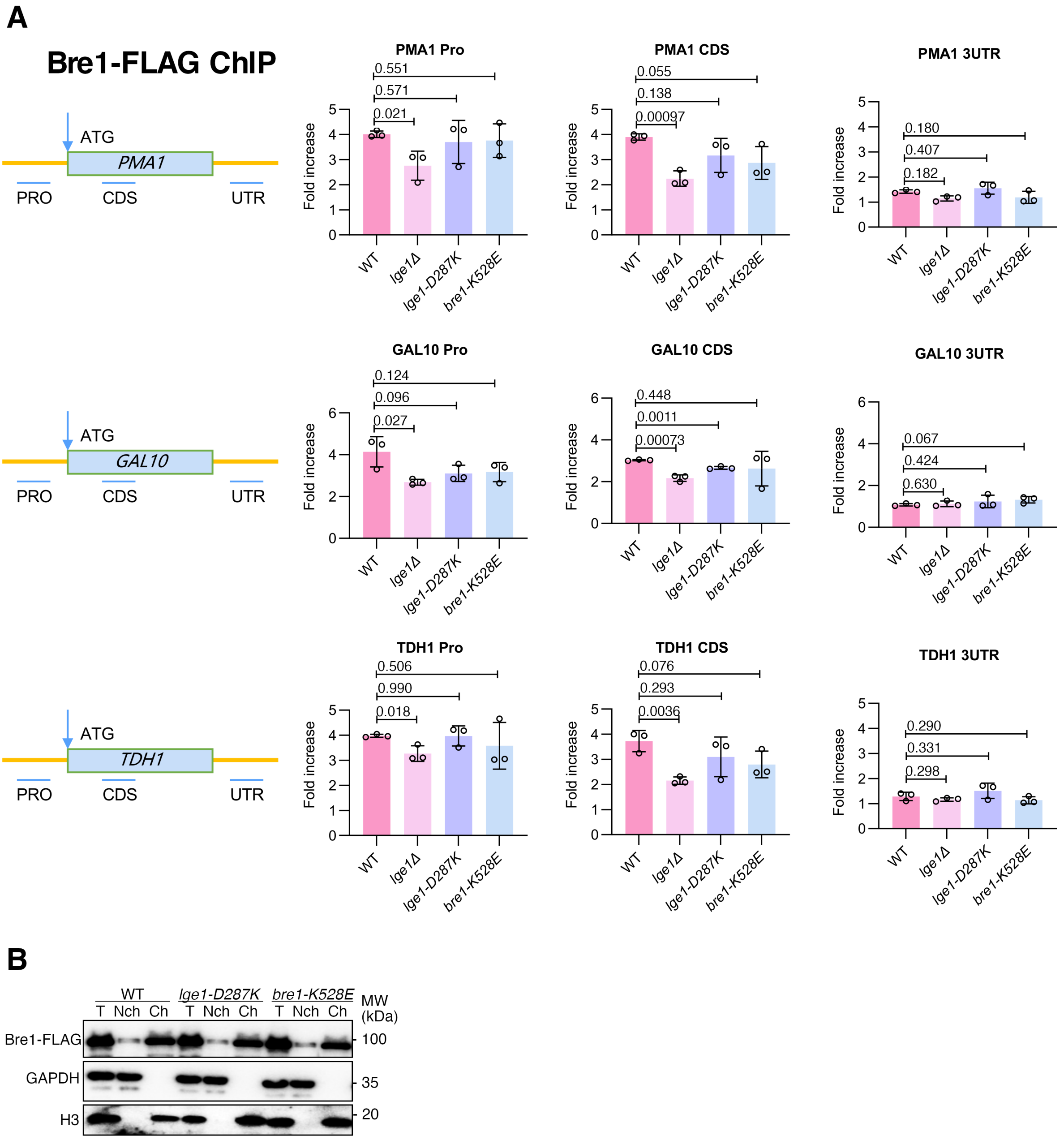

Figure S13 The Bre1-Lge1 interface is not required for Bre1 binding to chromatin. (A) Effects of mutations *lge1-D287K* and *bre1-K528E* on Bre1 distribution across several actively transcribed genes. The average and standard deviation of three independent ChIP experiments for Bre1 (hollow dots) are presented. The p-values are derived from the two-tailed Student's t-test. (B) Effects of mutations *lge1-D287K* and *bre1-K528E* on Bre1 binding to chromatin. T, total cell extract; Nch, non-chromatin faction; Ch, chromatin fraction.

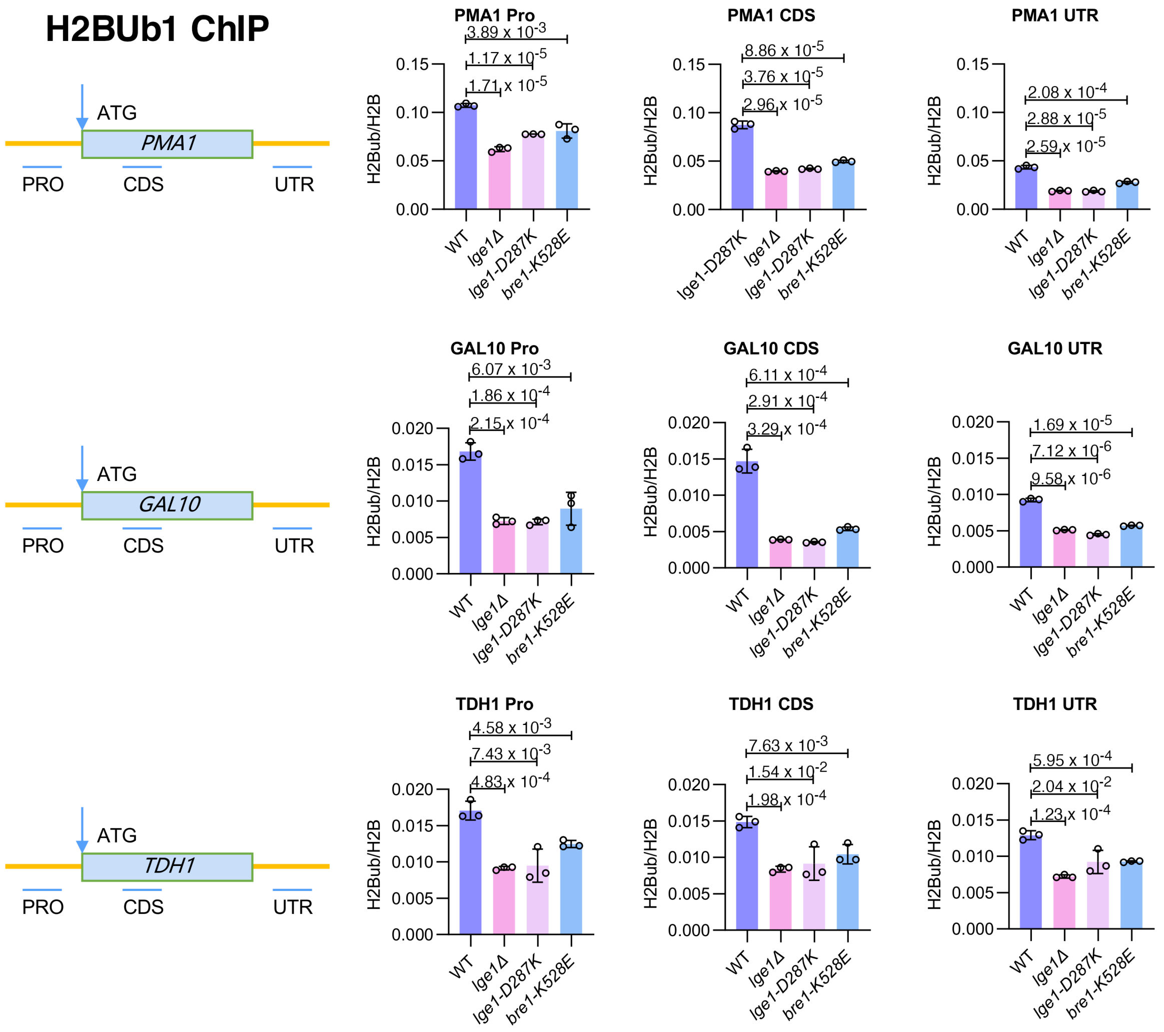

Figure S14 Effects of mutations *lge1-D287K* and *bre1-K528E* on H2BUb1 levels across several actively transcribed genes. The average and standard deviation of three independent ChIP experiments for H2BUb1 (hollow dots) are presented. The p-values are derived from the two-tailed Student's t-test.

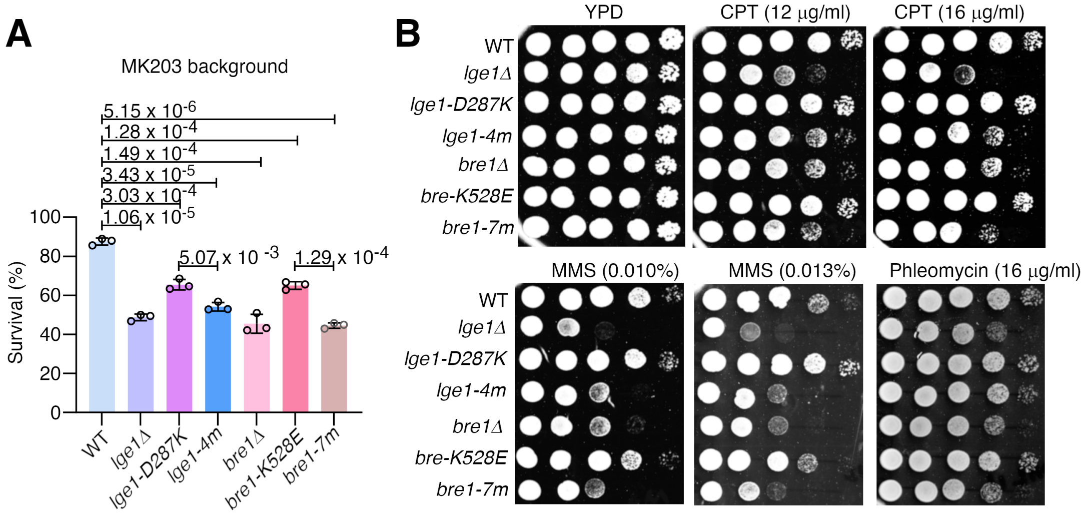

Figure S15 *In vivo* effects of combined mutations in *bre1* and *lge1*. (A) Ectopic recombination experiments probing the *in vivo* effects of the combined mutations on HR repair. The average and standard deviation of three independent experiments (hollow dots) are presented. The p-values are derived from the two-tailed Student's t-test. (B) Effects of the combined mutations on cell survival in the presence of DNA damaging agents. *Bre1-7m*, the K352E/R360E/D368K/K506E/K510E/K519E/K528E mutation in *BRE1*; *lge1-4m*, the D287K/E301K/E303K/R305E mutation in *LGE1*.

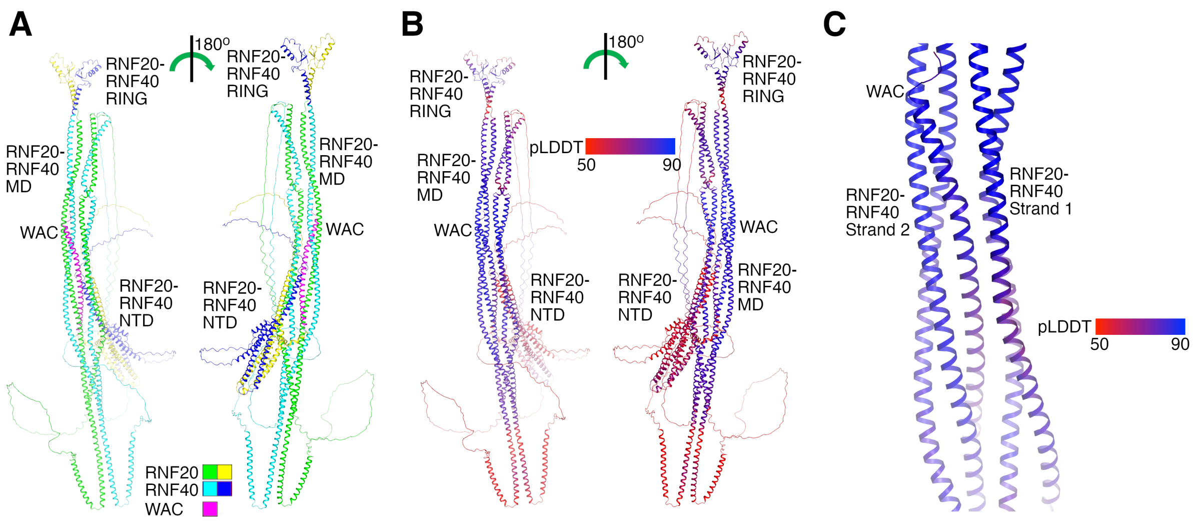

Figure S16 Structure prediction of the RNF20/RNF40 complex bound with WAC-CT. (A)-(B) The AlphaFold-predicted structure of the RNF20/RNF40 complex bound with WAC-CT. In panel (A), WAC, the RNF20 and RNF40 polypeptides in the NTD, MD and RING domains are presented in different colors. In panel (B), the predicted structure is colored according to the pLDDT scores. (C) WAC-CT and its binding regions in RNF-MD. The structure is colored according to the pLDDT scores.

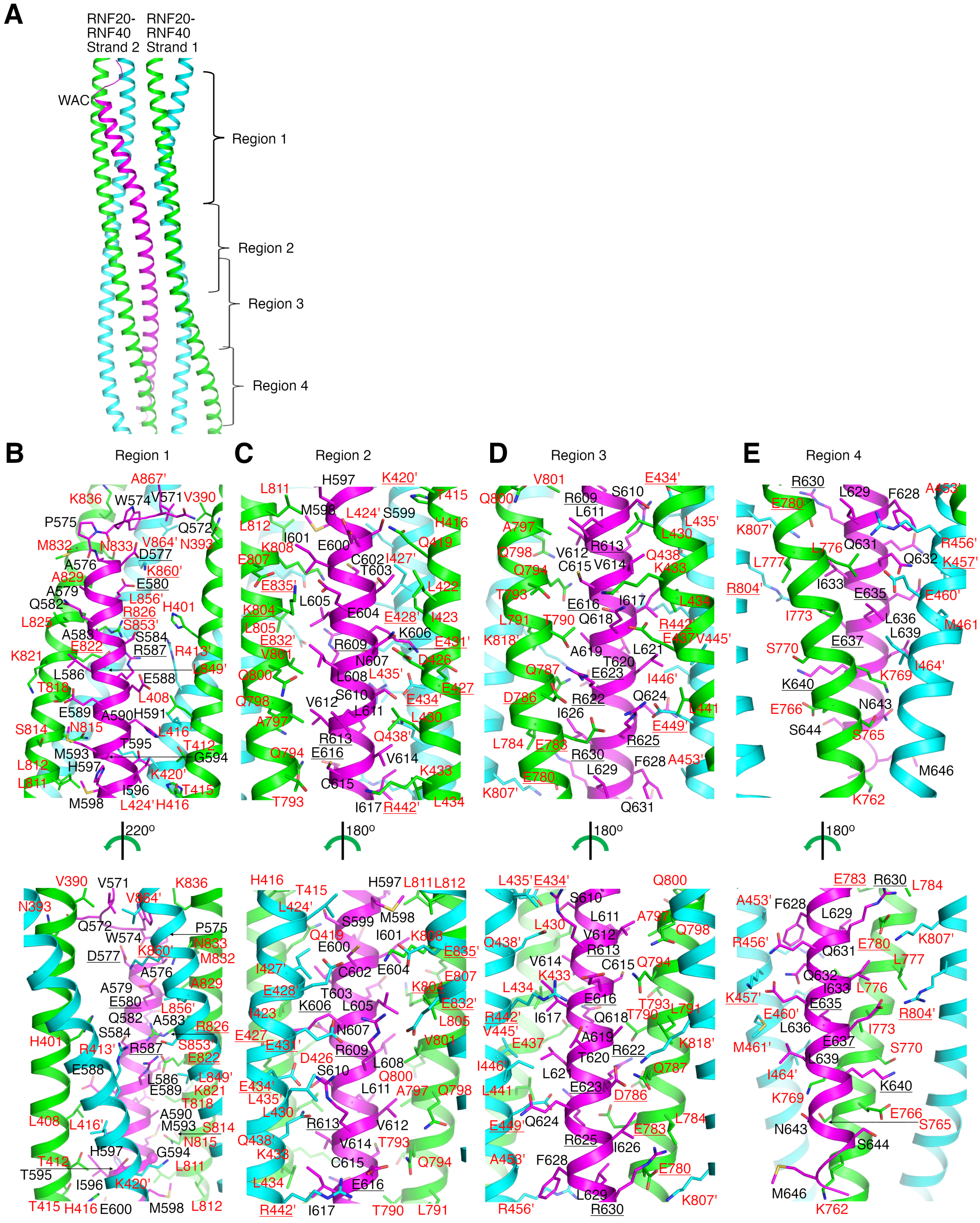

Figure S17 Interactions at the predicted RNF20/RNF40-WAC interface. The interface is divided into regions 1-4 (A) and interactions mediated by these regions are shown in panels (B)-(E). Residues in the RNF20/RNF40 complex and WAC are labelled in red and black, respectively. The ’ sign indicates residues in RNF40. Residues mediating electrostatic interactions are indicated by underlined labels.

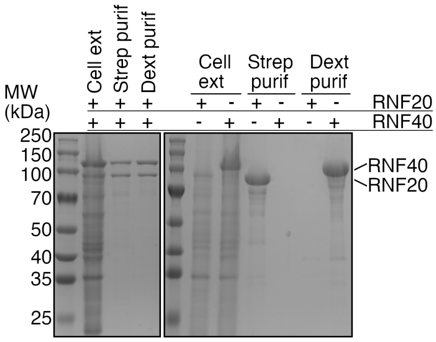

Figure S18 SDS-PAGE analysis for the purification of the RNF-MD complex. The gel on the left shows purification of the RNF-MD complex by sequential steps with strep-tactin and dextrin resins. The gel on the right shows purification of RNF20 or RNF40 polypeptides with strep-tactin or dextrin resins. Cell ext, cell extract; strep purif, purification with the strep-tactin resin; dext purif, purification with the dextrin resin.

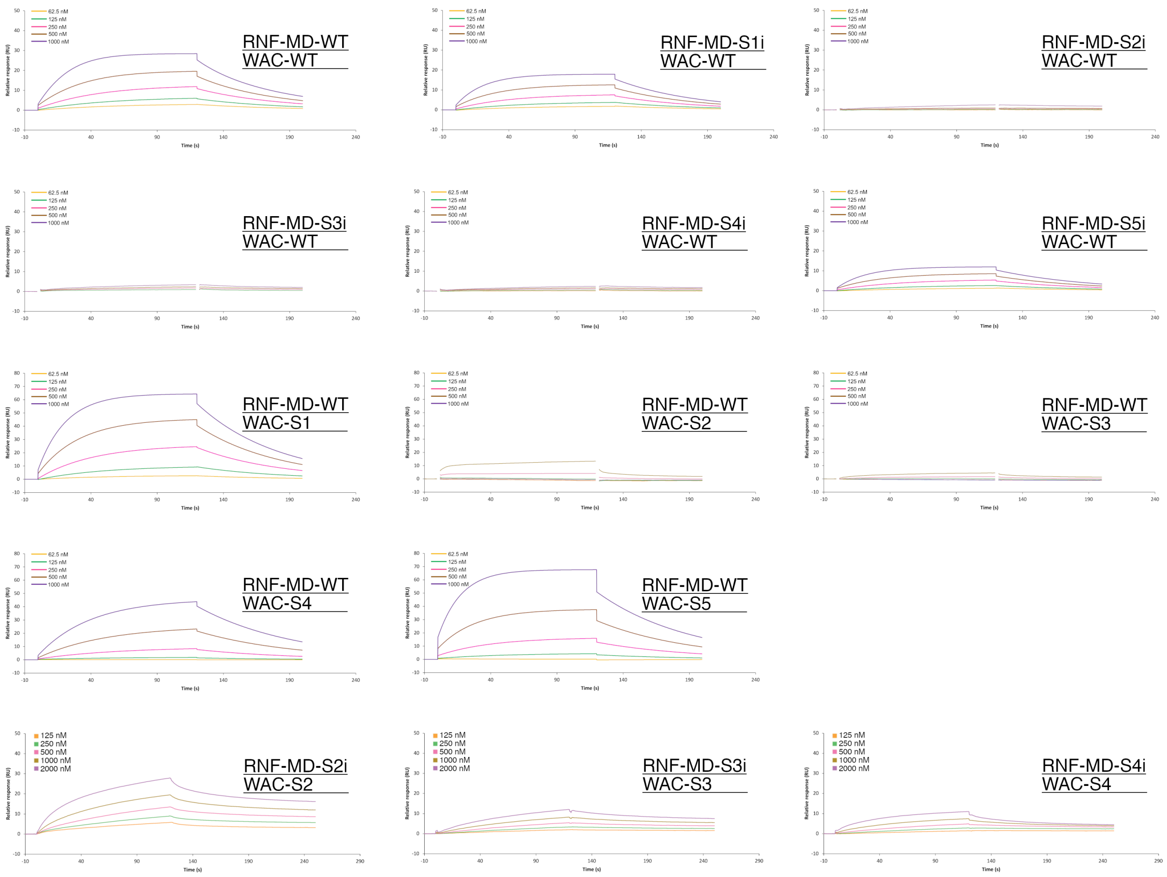

Figure S19 SPR experiments probing interactions at the predicted interface between the RNF20/RNF40 complex and WAC.

References

1. Shen, M., Dhingra, N., Wang, Q., Cheng, C., Zhu, S., Tian, X., Yu, J., Gong, X., Li, X., Zhang, H. *et al.* (2021) Structural basis for the multi-activity factor Rad5 in replication stress tolerance. *Nat Commun*, **12**, 321.
2. Lee, S.E., Moore, J.K., Holmes, A., Umezu, K., Kolodner, R.D. and Haber, J.E. (1998) Saccharomyces Ku70, mre11/rad50 and RPA proteins regulate adaptation to G2/M arrest after DNA damage. *Cell*, **94**, 399-409.
3. Ira, G., Malkova, A., Liberi, G., Foiani, M. and Haber, J.E. (2003) Srs2 and Sgs1-Top3 suppress crossovers during double-strand break repair in yeast. *Cell*, **115**, 401-411.
4. Zhao, X., Chabes, A., Domkin, V., Thelander, L. and Rothstein, R. (2001) The ribonucleotide reductase inhibitor Sml1 is a new target of the Mec1/Rad53 kinase cascade during growth and in response to DNA damage. *EMBO J*, **20**, 3544-3553.
